# The small molecule CA140 inhibits the neuroinflammatory response in wild-type mice and a mouse model of AD

**DOI:** 10.1186/s12974-018-1321-3

**Published:** 2018-10-11

**Authors:** Ju-Young Lee, Jin Han Nam, Youngpyo Nam, Hye Yeon Nam, Gwangho Yoon, Eunhwa Ko, Sang-Bum Kim, Mahealani R Bautista, Christina C Capule, Takaoki Koyanagi, Geoffray Leriche, Hwan Geun Choi, Jerry Yang, Jeongyeon Kim, Hyang-Sook Hoe

**Affiliations:** 1 Department of Neural Development and Disease, Korea Brain Research Institute (KBRI), 61 Cheomdan-ro, Dong-gu, Daegu, 41068 South Korea; 2 New Drug Development Center, Daegu-Gyeongbuk Medical Innovation Foundation, 80 Cheombok-ro, Dong-gu, Daegu, 41061 South Korea; 3 Department of Chemistry and Biochemistry, University of California, San Diego, La Jolla, CA 92093-0358 USA

**Keywords:** Alzheimer’s disease, Neuroinflammation, D1R, ERK, STAT3, LPS, CA140

## Abstract

**Background:**

Neuroinflammation is associated with neurodegenerative diseases, including Alzheimer’s disease (AD). Thus, modulating the neuroinflammatory response represents a potential therapeutic strategy for treating neurodegenerative diseases. Several recent studies have shown that dopamine (DA) and its receptors are expressed in immune cells and are involved in the neuroinflammatory response. Thus, we recently developed and synthesized a non-self-polymerizing analog of DA (CA140) and examined the effect of CA140 on neuroinflammation.

**Methods:**

To determine the effects of CA140 on the neuroinflammatory response, BV2 microglial cells were pretreated with lipopolysaccharide (LPS, 1 μg/mL), followed by treatment with CA140 (10 μM) and analysis by reverse transcription-polymerase chain reaction (RT-PCR). To examine whether CA140 alters the neuroinflammatory response in vivo, wild-type mice were injected with both LPS (10 mg/kg, intraperitoneally (i.p.)) and CA140 (30 mg/kg, i.p.), and immunohistochemistry was performed. In addition, familial AD (5xFAD) mice were injected with CA140 or vehicle daily for 2 weeks and examined for microglial and astrocyte activation.

**Results:**

Pre- or post-treatment with CA140 differentially regulated proinflammatory responses in LPS-stimulated microglia and astrocytes. Interestingly, CA140 regulated D1R levels to alter LPS-induced proinflammatory responses. CA140 significantly downregulated LPS-induced phosphorylation of ERK and STAT3 in BV2 microglia cells. In addition, CA140-injected wild-type mice exhibited significantly decreased LPS-induced microglial and astrocyte activation. Moreover, CA140-injected 5xFAD mice exhibited significantly reduced microglial and astrocyte activation.

**Conclusions:**

CA140 may be beneficial for preventing and treating neuroinflammatory-related diseases, including AD.

**Electronic supplementary material:**

The online version of this article (10.1186/s12974-018-1321-3) contains supplementary material, which is available to authorized users.

## Background

Increasing evidence indicates a critical role of the immune system in neurodegenerative diseases such as Alzheimer’s disease (AD) [1]. Abnormal glial activation in patients with neurodegenerative diseases may be a hallmark diagnostic feature of these diseases, particularly AD [2]. Neuroinflammation or inflammation of the central nervous system (CNS) is mainly mediated by the activation of microglia [1]. In addition to releasing various neurotrophic factors that support neuronal cell survival and neurotoxic factors, activated microglia release proinflammatory cytokines such as interleukin-1β (IL-1β) and tumor necrosis factor-α (TNF-α) [3, 4]. The neuroinflammation induced by the release of these proinflammatory cytokines may eventually lead to neuronal cell death and synaptic dysfunction. Therefore, the elucidation of the regulation of glial activation and inactivation may provide a potential therapeutic strategy for treating neurodegenerative diseases.

Lipopolysaccharide (LPS) is a well-established stimulator that induces the activation of microglial cells and is widely used both in vivo and in vitro to induce neuroinflammation in animal models [5, 6]. The interaction between LPS and Toll-like receptor 4 (TLR4) activates inflammation-associated transcription factors [7] and the mitogen-activated protein kinase (MAPK) family [8, 9], which comprises at least three components: extracellular signal-regulated kinases (ERKs), c-Jun N-terminal kinase (JNK), and p38 MAPK. In addition, the association between LPS and TLR4 stimulates the release of both immune-related cytotoxic factors, including iNOS and COX-2, and proinflammatory cytokines (TNF-α, IL-1β, and IL-6) [10]. A chronic inflammatory response may be accompanied by amyloid beta (Aβ) production, and microglia have been identified near the Aβ plaques of AD patients [11, 12]. Aβ accumulation triggers AD pathogenesis through two mechanisms: neuronal apoptosis and glia-mediated inflammation leading to cell death [13]. Extracellular Aβ deposits in senile plaques trigger changes in glial reactivity and stimulate neuroinflammation. Thus, Aβ accumulation may lead to neuronal loss through the overproduction of reactive proinflammatory cytokines [14, 15]. CA140 is a chemically stable small-molecule analog of dopamine (DA) and is synthesized by acylation of the amine in DA with N-methylisatoic anhydride, which reduces the propensity of DA to undergo self-polymerization [16]. DA is a neurotransmitter that regulates a wide range of functions, including initiation of movement and learning and memory [17]. DA binds to several DA receptors, which are present on nearly all immune cells [18]. Activation of these receptors via DA or DA agonists modulates the activation, proliferation, and cytokine production of immune cells [19]. We therefore speculated that CA140 may also exhibit biological activity against the neuroinflammatory response.

In the present study, we examined whether CA140 regulates the neuroinflammatory response in vitro and in vivo*.* We discovered that CA140 reduced proinflammatory responses in LPS-stimulated BV2 microglial cells, primary microglial cells, and primary astrocytes. In addition, CA140 inhibited LPS-induced neuroinflammatory responses by inhibiting the dopamine D1 receptor (D1R)/ERK/STAT3 signaling pathways. Moreover, CA140 significantly decreased the activation of microglia and astrocytes in wild-type mice as well as a mouse model of AD. Taken together, our results indicate that CA140 is a potential therapeutic agent for treating and/or preventing neuroinflammation-related diseases, including AD.

## Methods

### Cell lines and culture conditions

BV2 microglial cells (a generous gift of Dr. Kyung-Ho Suk) or HEK cells (a generous gift of Dr. Hyung-Jun Kim) were maintained in high-glucose DMEM (Invitrogen, Carlsbad, CA, USA) with 5 or 10% fetal bovine serum (FBS, Invitrogen, Carlsbad, CA, USA) in a 5% CO_2_ incubator.

### Mouse primary microglial and astrocyte cultures

Mouse primary microglial and astrocyte cultures were prepared from mixed glial cultures as previously described [20]. Briefly, whole brains of post-natal 1-day-old C57BL/6 mice were chopped and mechanically disrupted using a 70-μm nylon mesh. The cells were seeded in 75 T culture flasks and grown in low-glucose DMEM supplemented with 10% FBS, 100 unit/mL penicillin, and 100 μg/mL streptomycin. The culture medium was changed after 7 days and every 3 days thereafter. After 14 days, mixed primary glial cells were obtained for use in subsequent experiments. To obtain mouse primary astrocytes, mixed glial cells were cultured with shaking at 250 rpm overnight. The next day, the culture medium was discarded, and the cells were washed three times with PBS. The cells were dissociated using trypsin-EDTA and collected by centrifugation at 1200 rpm for 10 min. Primary astrocytes were maintained in low-glucose DMEM supplemented with 10% FBS and penicillin-streptomycin. To obtain mouse primary microglial cells, mixed primary glial cells were incubated with trypsin solution (0.25% trypsin, 1 mM EDTA in Hank’s balanced salt solution) diluted 1:4 in serum-free DMEM media [21]. After the mouse primary astrocyte layer was fully detached, low-glucose DMEM containing 10% FBS was added, the supernatant was aspirated, and the remaining primary microglial cells were used for experiments.

### Rat primary microglial and astrocyte cultures

Rat primary mixed glial cells were cultured from the cerebral cortices of 1-day-old Sprague Dawley rats. Briefly, the cortices were triturated into single cells in high-glucose DMEM containing 10% FBS/penicillin-streptomycin solution (5000 units/mL penicillin, 5 mg/mL streptomycin, Corning, Mediatech Inc., Manassas, VA, USA) and plated into 75 T culture flasks (0.5 hemisphere/flask) for 2 weeks. To harvest rat primary microglial cells, the plate was shaken continuously at 120 rpm for 2 h to facilitate microglial detachment from the plate. The fluid medium was subsequently collected and centrifuged at 1500 rpm for 15 min, and the cell pellets were resuspended to plate 1 × 10^5^ cells per well. The remaining cells in the flask were harvested using 0.1% trypsin to obtain rat primary astrocytes. These rat primary astrocytes and primary microglial cells were cultured in 12-well plates (35 mm) pre-coated with poly-D-lysine (Sigma).

### Wild-type mice

All experiments were performed in accordance with the approved animal protocols and guidelines established by the Korea Brain Research Institute (IACUC-2016-0013). C57BL6/N mice were purchased from Orient-Bio Company. Male C57BL6/N mice (8 weeks, 25–30 g) were housed in a pathogen-free facility with 12 h of light and dark per day at an ambient temperature of 22 °C. To determine if pretreatment with CA140 alters LPS-induced neuroinflammation, wild-type mice were intraperitoneally (i.p.) administered CA140 (30 mg/kg) or vehicle (10% DMSO) daily for 3 days and subsequently injected with LPS (Sigma, *Escherichia coli*, 10 mg/kg, i.p.) or PBS. After 3 h, immunostaining was performed with anti-IbaI or anti-GFAP antibodies. To examine whether post-treatment with CA140 regulates LPS-induced neuroinflammatory responses, wild-type mice were injected with LPS (10 mg/kg/day, i.p.) or PBS, followed 30 min later by injection with CA140 (30 mg/kg, i.p., twice with an interval of 1 h, followed 30 min later by a third injection) or vehicle (10% DMSO, i.p.). Immunohistochemistry was then performed with anti-Iba-1 and anti-GFAP antibodies.

### Familial AD (5xFAD) mice

F1 generation 5xFAD mice (stock number 008730, B6SJL-Tg APPSwFlLon, PSEN1*M146 L*L286V6799Vas/Mmjax) were purchased from The Jackson Laboratory. 5xFAD mice overexpress two mutant human proteins: APP (695) with KM670/671NL (Swedish), I716V (Florida), and V717I (London) FAD mutations and PS1 with M146 L and L286 V FAD mutations. To examine the effects of CA140 on the neuroinflammatory response in a mouse model of AD, 5xFAD mice were injected with CA140 (30 mg/kg, i.p.) or vehicle (10% DMSO, i.p.) daily for 2 weeks, and immunohistochemistry was conducted with anti-Iba-1 or anti-GFAP antibodies. The animal groups were randomized for all experiments. Data were analyzed in a semi-automated manner using ImageJ software and confirmed by an independent researcher who did not participate in the current experiments. Only male mice were used for this study because the pathology of 5xFAD female mice is more severe than that of male mice, leading to huge variations in in vivo experiments.

### Synthesis of CA140

*N*-Methylisatoic anhydride (19.6 mg, 110 μmol) was added to 28.1 mg of 3,4-dimethoxyphenethylamine (155 μmol, 1.3 equiv) and 25 μL of triethylamine (339 μmol, 3 equiv) in dichloromethane (DCM). The reaction mixture was stirred for 2 h at room temperature and overnight at 20 °C. The mixture was warmed to room temperature, and a vacuum was subsequently applied to remove volatile organics. The amide product was purified by column chromatography (7% ethyl acetate in DCM; Rf = 0.27) to provide 31.5 mg of *N*-(3,4-dimethoxyphenethyl)-2-(methylamino)benzamide as a white solid (91% isolated yield). ^1^H-NMR (400 MHz, CDCl_3_) δ ppm = 7.30 (t, 1H, *J* = 7.7 Hz, Ar-H), 7.20 (d, 1H, *J* = 7.7 Hz, Ar-H), 6.82 (d, 1H, *J* = 8.1 Hz, Ar-H), 6.74 (m 2H, Ar-H), 6.55 (t, 1H, *J* = 7.5 Hz, Ar-H), 3.86 (s, 3H, OCH_3_), 3.83 (s, 3H, OCH_3_), 3.63 (t, 1H, *J* = 6.7 Hz, -HCH-), 3.61 (t, 1H, *J* = 6.7 Hz, -HCH-), 2.84 (s, 3H, CH_3_), 2.84 (t, 2H, *J* = 6.8 Hz, CH_2_). ^13^C-NMR (100 MHz, CDCl_3_) δ ppm = 169.6, 150.0, 148.9, 147.6, 132.7, 131.4, 127.0, 120.6, 115.4, 114.8, 111.9, 111.4, 111.3, 55.8, 55.7, 40.9, 35.1, 29.8.

*N*-(3,4-Dimethoxyphenethyl)-2-(methylamino)benzamide (9.2 mg, 29.3 μmol) was dissolved in 0.5 mL of anhydrous DCM, and 120 μL of 1 M BBr_3_ in DCM (120 μmol, 4 equiv) was added. The reaction was stirred overnight under an inert atmosphere. Excess methanol was added to the mixture, and volatile organics were removed in vacuo. The addition and removal of methanol was repeated at least three times to remove boric acid as methyl borate. The target compound, *N*-(3,4-dihydroxyphenethyl)-2-(methylamino)benzamide (CA140), was obtained in quantitative yield. ^1^H-NMR (400 MHz, CD_3_OD) δ ppm = 7.91 (d, 1H, *J* = 8.0 Hz, Ar-H), 7.89 (t, 1H, *J* = 7.6 Hz, Ar-H), 7.74 (m, 2H, *J* = 7.6 Hz, Ar-H), 6.67 (d 2H, *J* = 9.6 Hz Ar-H), 6.56 (d, 1H, *J* = 8.0 Hz, Ar-H), 3.60 (t, 2H, *J* = 7.1 Hz, CH_2_), 3.01 (s, 3H, NCH_3_), 2.79 (t, 1H, *J* = 7.0 Hz, CH_2_). LC-MS ESI positive mode *m/z* [M + H]^+^ = 287.11 (calculated = 287.13).

### Brain-to-plasma ratio in ICR (Institute for Cancer Research) mice

ICR mice (*n* = 3) were dosed with CA140 dissolved in DMSO/Tween-80/saline (10:5:85%) via a single intravenous administration (10 mg/kg). Blood was collected by cardiac puncture at 5 min and then centrifuged to isolate plasma. The brain was collected at 5 min and homogenized in PBS after washing with fresh PBS. The concentrations of CA140 in the plasma and brain were determined by LC-MS/MS. The LC-MS/MS system comprised a Nexera XR HPLC system (Shimadzu Co., Kyoto, Japan) coupled to a TSQ Vantage triple quadrupole mass spectrometer equipped with Xcalibur version 1.1.1 (Thermo Fisher Scientific Inc., Waltham, MA, USA).

### Stability studies of CA140 and dopamine in vitro

To examine the stability of CA140 in vitro, samples were generated from stock solutions (30 mM in DMSO) of either dopamine (DA, as a control for CA140) or CA140 by dilution in 1 mL of preheated PBS buffer to yield final concentrations of 500 μM. The solutions were incubated at 37 °C in a thermoblock, and the concentration of CA140 or DA was followed over time in triplicate. Aliquots (50 μL) were taken at 0, 1, 2, 4, 6, 8, and 22 h and added to 200 μL of acetonitrile. The samples were mixed by vortexing for 30 s and then centrifuged at 4 °C for 15 min at 14,000 rpm. The clear supernatants were diluted in PBS (2-fold) and analyzed by HPLC at 254 and 280 nm for CA140 and DA, respectively.

### Antibodies and inhibitors

The following primary antibodies were used throughout this study: rat anti-mouse CD11b (1:400, Abcam), rabbit anti-F-actin (1:1000, Abcam), rabbit anti-COX-2 (1:1000, Abcam), rabbit anti-IL-1β (1:200, Abcam), rabbit anti-GFAP (1:5000, Neuromics), rabbit anti-Iba-1 (1:1000, Wako), goat anti-Iba-1 (1:500, Wako), rabbit anti-AKT (1:1000, Santa Cruz), rabbit anti-p-AKT (Ser473, Thr308) (1:1000, Cell Signaling), rabbit anti-ERK (1:1000, Santa Cruz), rabbit anti-p-ERK (Thr42/44) (1:1000, Cell Signaling), rabbit anti-STAT3 (1:1000, Cell Signaling), rabbit anti-p-STAT3 (Ser727, Abcam), mouse anti-PCNA (1:1000, Santa Cruz), rabbit anti-D2R (1:1000, Abcam), and rabbit anti-D1R (1:1000, Millipore) antibodies. We used the following small molecules: D1R antagonists (LE300, 10 μM, Sigma-Aldrich; SCH23390, 30 μM, Tocris), D1R agonist (A77636 hydrochloride, 10 nM, Tocris), D2R antagonist (eticlopride hydrochloride, 100 nM, Sigma-Aldrich), a STAT3 inhibitor (S3I-201, 50 μM, Sigma-Aldrich), and an ERK inhibitor (PD98059, 10 μM, Millipore).

### MTT assay

BV2 microglial cell viability was assessed using the 3-(4,5-dimethylthiazol-2-yl)-2,5-diphenyltetrazolium bromide (MTT) assay. BV2 microglial cells were seeded in 96-well plates and treated with various concentrations of CA140 (1–50 μM) or vehicle (1% DMSO) for 24 h in the absence of FBS. The cells were subsequently treated with 0.5 mg/mL MTT and incubated for 3 h at 37 °C in a 5% CO_2_ incubator. The absorbance was read at 580 nm.

### Reverse transcription-polymerase chain reaction (RT-PCR)

Total RNA was extracted from cells using TRIzol (Invitrogen) following the manufacturer’s instructions. Total RNA was reverse transcribed into cDNA using a Superscript cDNA Premix Kit II with oligoDT (GeNetBio, Korea), and RT-PCR was performed using Prime Taq Premix (GeNetBio, Korea). RT-PCR products were separated by electrophoresis on 1.5% agarose gels with Eco Dye (1:5000, Korea) and photographed. Images were analyzed using ImageJ (NIH) and Fusion (Korea).

### Immunocytochemistry

BV2 microglial cells were fixed in ice-cold methanol for 8 min, washed three times with 1 × PBS, and incubated with CD11b and COX-2 or CD11b and IL-1β antibodies in GDB buffer (0.1% gelatin, 0.3% Triton X-100, 16 mM sodium phosphate pH 7.4, and 450 mM NaCl) overnight at 4 °C. The next day, the cells were washed three times with 1 × PBS and incubated with the following secondary antibodies for 1 h at room temperature: Alexa Fluor 488 and Alexa Fluor 555 (1:200, Molecular Probes, USA). Images were obtained on a single plane using a confocal microscope (Nikon, Japan) and analyzed using ImageJ software.

### Immunohistochemistry and immunofluorescence

Animals were perfused and fixed with 4% paraformaldehyde (PFA) solution, and brain tissues were flash-frozen and dissected using a cryostat (35-mm-thick sections). Each brain section was processed for immunofluorescence or immunohistochemical staining. For immunofluorescence staining, sections were rinsed in PBS and incubated with rabbit anti-Iba-1 (1:1000, Wako, Japan) for microglia or rabbit anti-GFAP (1:5000, Neuromics) for astrocytes. Antibodies were diluted in 0.5% bovine serum albumin (BSA) and incubated at 4 °C overnight. The following day, tissues were rinsed with 0.5% BSA and incubated with Alexa Fluor 555-conjugated anti-rabbit IgG (1:200, Molecular Probes) for 1 h at room temperature. The tissues were subsequently mounted on a gelatin-coated cover glass and covered with DAPI-containing mounting solution (Vector Laboratories). Images of the stained tissues were captured using confocal microscopy (TI-RCP, Nikon).

For immunohistochemistry, sections were permeabilized for 1 h in PBS with 0.2% Triton X-100 and 1% BSA at room temperature. The sections were then incubated with primary antibodies at 4 °C overnight. The next day, the tissues were washed three times with 0.5% BSA and incubated with biotin-conjugated anti-rabbit antibody (1:400, Vector Laboratories) for 1 h at room temperature. After rinsing with 0.5% BSA, the sections were incubated for 1 h at room temperature in avidin-biotin complex solution (Vector Laboratories, Burlingame, CA), followed by rinsing three times in 0.1 M phosphate buffer (PB). The signal was detected by incubating the sections in 0.5 mg/mL 3,3′-diaminobenzidine (DAB, Sigma-Aldrich) in 0.1 M PB containing 0.003% H_2_O_2_. The sections were rinsed in 0.1 M PB and mounted on gelatin-coated slides, and images were obtained under a bright-field microscope (Leica).

### Enzyme-linked immunosorbent assay (ELISA)

To measure the effects of pre- or post-treatment with CA140 on IL-1β, an enzyme-linked immunosorbent assay (ELISA) was performed. Briefly, BV2 microglial cells were treated with LPS (100 ng/mL) or PBS for 30 min, followed by treatment with CA140 (10 μM) or vehicle (1% DMSO). IL-1β ELISA was then performed using the conditioned medium. Mouse IL-1β ELISA kits (ELISA development reagents; R&D Systems, Minneapolis, MN) were used according to the manufacturer’s recommendations. Recombinant mouse IL-1β protein (R&D Systems) was used as a standard. The absorbance of the samples was measured at 450 nm using a microplate reader (BMG Labtech, Offenburg, Germany).

### Griess assay

To examine the effects of CA140 on nitrite (NO) production, the Griess assay was performed. BV2 microglial cells were incubated with CA140 (10 μM) or vehicle (1% DMSO) for 30 min, followed by treatment with LPS (100 ng/mL) or PBS for 23.5 h. The conditioned medium was mixed with Griess reagent (0.1% *N*-(1-naphthyl)ethylenediamine dihydrochloride and 1% sulfanilamide in 2% phosphoric acid) in 96-well plates and incubated at room temperature for 5 min. The absorbance was measured at 540 nm using a microplate reader, and the level of nitrite was analyzed against a standard curve of sodium nitrite.

### Western blotting

Cells were lysed using RIPA buffer containing protease and phosphatase inhibitor tablets (Roche, USA). Western blot analysis was performed as previously described [22]. Images were analyzed using Fusion software or ImageJ.

### Cytosolic and nuclear fractionation

BV2 microglial cells were lysed in cytosolic fractionation buffer (10 mM HEPES pH 8.0, 1.5 mM MgCl_2_, 10 mM KCl, 0.5 mM DTT, 300 mM sucrose, 0.1% NP-40, and 0.5 mM PMSF). After 5 min, the cell lysates were centrifuged at 10,000 rpm at 4 °C for 1 min, and the supernatant was stored as the cytosolic fraction. The pellet was lysed in nuclear fractionation buffer (10 mM HEPES pH 8.0, 20% glycerol, 100 mM KCl, 100 mM NaCl, 0.2 mM EDTA, 0.5 mM DTT, and 0.5 mM PMSF) on ice for 15 min, followed by centrifugation at 10,000 rpm at 4 °C for 15 min. The cytosolic and nuclear fractions were analyzed by western blot as previously described [23].

### Statistical analyses

All data were analyzed using a two-tailed *T* test or ANOVA with GraphPad Prism 4 software. Post hoc analyses were performed using Tukey’s multiple comparison test with significance set at *p* < 0.05. Data are presented as the mean ± S.E.M. (*p < 0.05, **p < 0.01, ***p < 0.001).

## Results

### CA140 has no cytotoxicity in BV2 microglial cells up to 25 μM

**Fig. 1:**
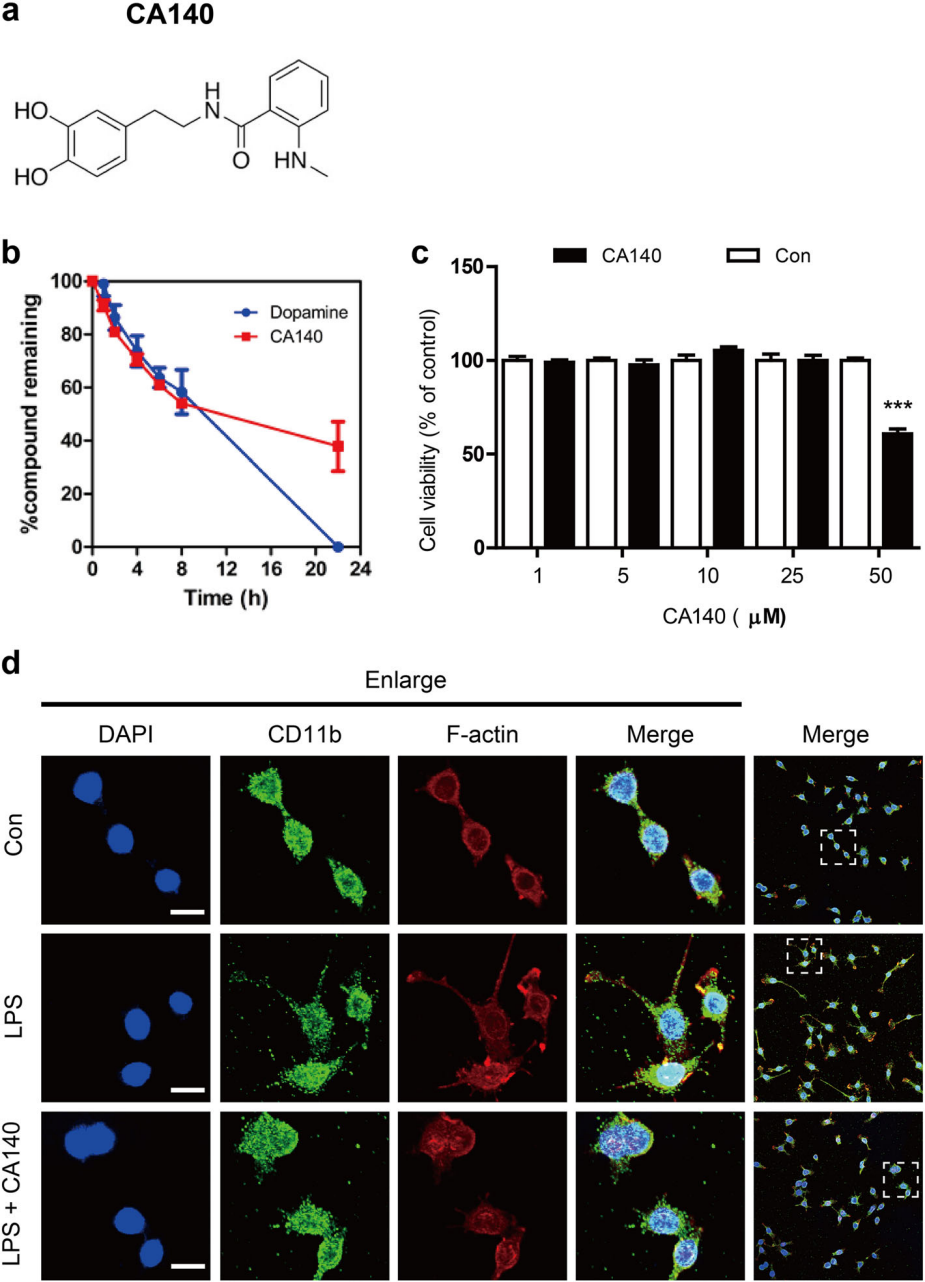
Concentrations of CA140 up to 25 μM were not toxic in BV2 microglial cells. **a** Structure of CA140. **b** Stability studies of CA140 and dopamine (DA) in vitro. **c** BV2 microglial cells were treated with vehicle (1% DMSO) or CA140 at various concentrations (1, 5, 10, 25, or 50 μM) for 24 h, and cell viability was measured (*n* = 8 for each dose). **d** BV2 microglial cells were pretreated with LPS (1 μg/mL) or PBS for 30 min, followed by treatment with vehicle (1% DMSO) or CA140 (10 μM) for 6 h and immunostaining with anti-CD11b and anti-F-actin antibodies. ***p < 0.001

We recently synthesized CA140 formally by benzoylation of dopamine (DA) with *N*-Methylisatoic anhydride (Fig. 1a) and examined whether the newly developed CA140 crosses the blood-brain barrier. In a pharmacokinetic study, we determined that the relevant brain-to-plasma ratio was 1.91 ± 0.22 (brain concentration, 2310–4457 ng/mL), which indicates a high brain distribution of CA140 (data not shown). In addition, we measured the stability of CA140 in vitro and found that the rates of disappearance of CA140 and DA (as a control for CA140) were comparable within the first 8 h. However, after 22 h, more than 40% of CA140 remained in solution, whereas DA was no longer detectable (Fig. 1b).

To examine the effects of CA140 on the LPS-induced neuroinflammatory response, we initially assessed the cytotoxicity of CA140 in BV2 microglial cells. BV2 microglial cells were treated with vehicle (1% DMSO) or CA140 (1, 5, 10, 25, or 50 μM) for 24 h, and MTT assays were performed. CA140 did not affect cell viability up to a concentration of 25 μM; however, CA140 exhibited some toxicity at 50 μM in BV2 microglial cells (Fig. 1c).

We then assessed whether CA140 can alter cell morphology in LPS-stimulated BV2 microglial cells. BV2 microglial cells were pretreated with LPS (1 μg/mL) or PBS for 30 min and treated with vehicle (1% DMSO) or CA140 (10 μM) for 5.5 h. After 6 h, BV2 microglial cells were fixed and immunostained with anti-CD11b and anti-F-actin antibodies. LPS treatment produced aberrant cell morphology of BV2 microglial cells, such as thin fibroblast-like processes from the cell body (Fig. 1d, middle panel). However, treatment with LPS followed by CA140 appeared to rescue this abnormal cell morphology (Fig. 1d, lower panel).

### CA140 reduces proinflammatory cytokine levels in LPS-stimulated BV2 microglial cells

**Fig. 2:**
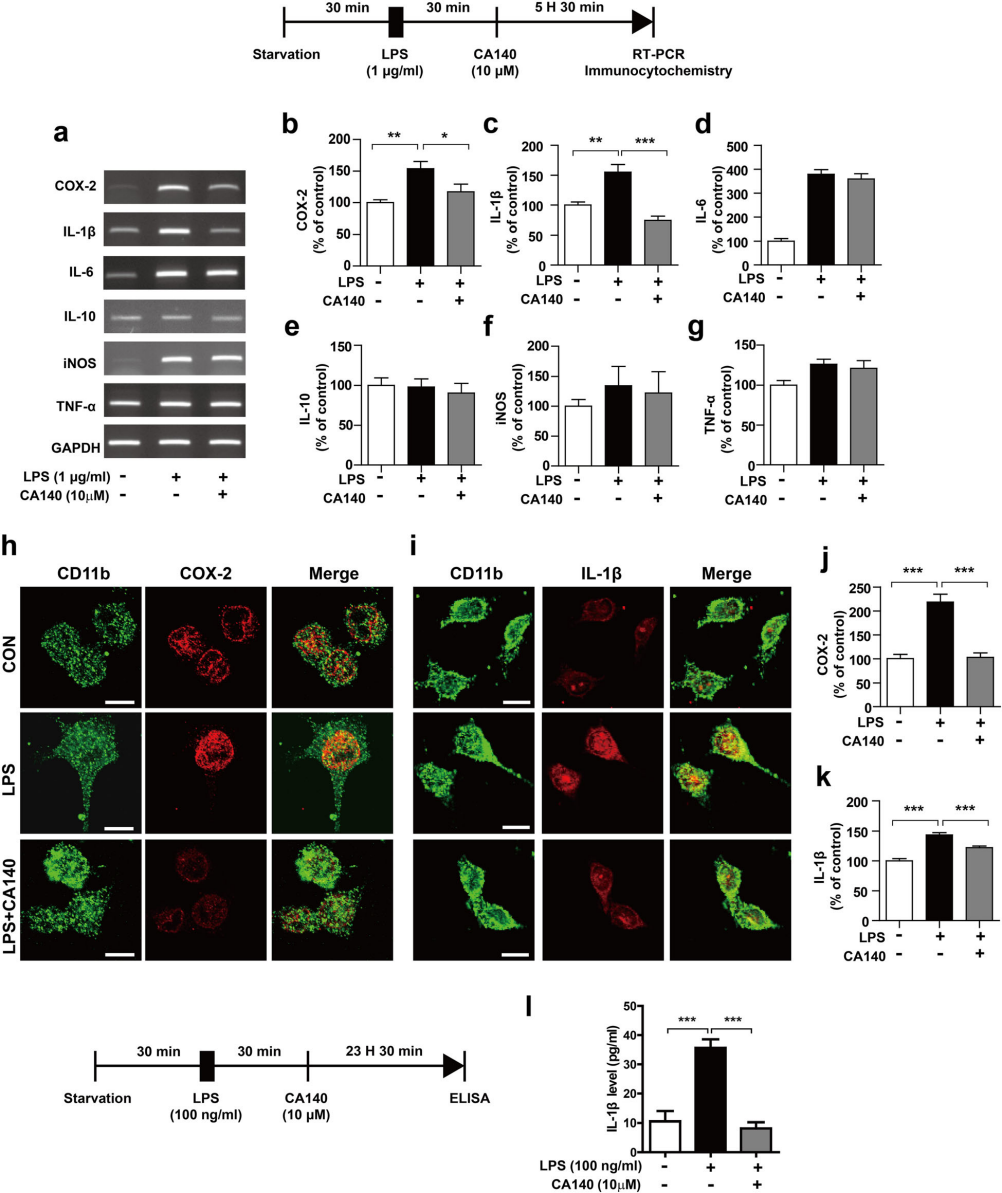
Pretreatment with LPS followed by CA140 treatment decreased LPS-induced proinflammatory cytokine levels. **a**–**g** BV2 microglial cells were pretreated with LPS (1 μg/mL) or PBS for 30 min, followed by treatment with vehicle (1% DMSO) or CA140 (10 μM) for 5.5 h. Total RNA was then isolated, and proinflammatory cytokine levels were measured by RT-PCR (COX-2: con, *n* = 17; LPS, *n* = 17; LPS + CA140, *n* = 17; IL-1β: con, *n* = 8; LPS, *n* = 8; LPS + CA140, *n* = 8; IL-6, IL-10, iNOS, and TNF-alpha: con, *n* = 4; LPS, *n* = 4; LPS + CA140, *n* = 4). **h**, **j** BV2 microglial cells were pretreated with LPS (1 μg/mL) or PBS for 30 min, followed by treatment with vehicle (1% DMSO) or CA140 (10 μM) for 5.5 h and immunostaining with anti-CD11b and anti-COX-2 antibodies (con, *n* = 74; LPS, *n* = 78; LPS + CA140, *n* = 84). **i**, **k** BV2 microglial cells were pretreated with LPS (1 μg/mL) or PBS for 30 min, followed by treatment with vehicle (1% DMSO) or CA140 (10 μM) for 5.5 h and immunostaining with anti-CD11b and anti-IL-1β antibodies (con, *n* = 314; LPS, *n* = 268; LPS + CA140, *n* = 234). **l** BV2 microglial cells were pretreated with LPS (100 ng/mL) or PBS for 30 min, followed by treatment with vehicle (1% DMSO) or CA140 (10 μM) for 23.5 h and measurement of IL-1β levels using IL-1β ELISA (con, *n* = 8; LPS, *n* = 8; LPS + CA140, *n* = 8). *p < 0.05, **p < 0.001, ***p < 0.0001

To determine whether post-treatment with CA140 reduces proinflammatory responses in LPS-stimulated BV2 microglial cells, cells were pretreated with LPS (1 μg/mL) or PBS for 30 min, followed by treatment with CA140 (5 or 10 μM) or vehicle (1% DMSO) for 5.5 h. Proinflammatory cytokine levels were then measured by RT-PCR. Interestingly, we observed that 5 μM CA140 only significantly decreased LPS-induced IL-1β mRNA levels (Additional file 1: Figure S1a–g). To determine if post-treatment with a higher concentration of CA140 could alter LPS-stimulated increase in proinflammatory cytokine levels, BV2 microglial cells were subjected to the same procedure but with 10 μM CA140, and proinflammatory cytokine levels were measured by RT-PCR. Post-treatment with 10 μM CA140 significantly reduced LPS-induced IL-1β and COX-2 mRNA levels in BV2 microglial cells (Fig. 2a–g). As a complementary study, BV2 microglial cells were treated with LPS (1 μg/mL) or PBS for 30 min, followed by treatment with vehicle (1% DMSO) or CA140 (10 μM) for 5.5 h and analysis by immunocytochemistry. Consistent with our findings above, post-treatment with CA140 also significantly reduced the levels of COX-2 and IL-1β in LPS-stimulated BV2 microglial cells (Fig. 2h–k). To further confirm these findings, we conducted an IL-1β ELISA assay. For this experiment, BV2 microglial cells were pretreated with LPS (100 ng/mL) or PBS for 30 min, followed by treatment with CA140 (10 μM) or vehicle (1% DMSO) for 23.5 h. The IL-1β ELISA assay was then performed. Consistent with the findings above, post-treatment with CA140 significantly decreased LPS-induced IL-1β levels compared with LPS treatment alone (Fig. 2l).

We then examined whether post-treatment with CA140 can further regulate LPS-induced proinflammatory cytokine levels in a longer treatment. BV2 microglial cells were pretreated with LPS (1 μg/mL) or PBS for 30 min, followed by treatment with CA140 (10 μM) or vehicle (1% DMSO) for 11.5 or 23.5 h. Proinflammatory cytokine levels were then measured by RT-PCR. Interestingly, post-treatment with CA140 significantly suppressed LPS-induced proinflammatory cytokine levels in a time-dependent manner (Additional file 1: Figure S2a–l).

We subsequently examined whether pretreatment with CA140 prevents LPS-induced neuroinflammatory responses. BV2 microglial cells were incubated with CA140 (5 μM) or vehicle (1% DMSO) for 30 min, followed by LPS (1 μg/mL) or PBS for 5.5 h. Proinflammatory cytokine levels were evaluated by RT-PCR. Pretreatment with 5 μM CA140 followed by LPS treatment did not decrease proinflammatory cytokine levels (Additional file 1: Figure S3a–g). However, pretreatment with 10 μM CA140 significantly reduced the mRNA levels of COX-2, IL-1β, and iNOS (Additional file 1: Figure S3h–n). In addition, pretreatment with CA140 at 10 μM significantly decreased LPS-induced IL-1β and NO levels as assessed by IL-1β ELISA or the Griess assay (Additional file 1: Figure S3o–p).

We then investigated whether pretreatment with CA140 further regulates LPS-induced proinflammatory cytokine levels in a longer treatment. BV2 microglial cells were incubated with CA140 (10 μM) or vehicle (1% DMSO) for 30 min, followed by LPS (1 μg/mL) or PBS for 11.5 or 23.5 h. Longer pretreatment with CA140 further reduced LPS-induced mRNA levels of the proinflammatory cytokines COX-2, IL-1β, and iNOS compared with pretreatment for 6 h (Additional file 1: Figure S4a–h). These results suggest that CA140 both reduces and prevents proinflammatory responses in LPS-induced BV2 microglial cells. Based on these findings, we selected 10 μM CA140 as our optimal working concentration for further experiments.

### CA140 reduces LPS-induced proinflammatory cytokine levels in primary microglial cells and primary astrocytes

Although BV2 microglial cells have been extensively used as an alternative model system for investigating microglial function in neuroinflammation [24], we aimed to examine whether pre- or post-treatment with CA140 modulates proinflammatory responses in different cell types, such as primary microglial cells or primary astrocytes. For these experiments, rat primary microglial or primary astrocyte cultures in high-glucose DMEM were used. Rat primary microglial cells were treated with LPS (1 μg/mL) or PBS for 30 min, followed by vehicle (1% DMSO) or CA140 (10 μM) for 5.5 h. Proinflammatory cytokine levels were measured by RT-PCR. Post-treatment with CA140 significantly decreased the mRNA levels of COX-2 and IL-1β in LPS-stimulated rat primary microglial cells (Additional file 1: Figure S5a–f) but failed to lower proinflammatory cytokine levels in LPS-induced rat primary astrocytes (Additional file 1: Figure S5g–l).

We subsequently examined whether pretreatment with CA140 differentially regulates LPS-induced proinflammatory responses in rat primary microglial cells and primary astrocytes. Rat primary microglial cells or primary astrocytes were pretreated with CA140 (10 μM) or vehicle (1% DMSO) for 30 min, followed by treatment with LPS (1 μg/mL) or PBS for 5.5 h. Proinflammatory cytokine levels were then measured by RT-PCR. Interestingly, pretreatment with CA140 reduced LPS-induced COX-2, IL-1β, iNOS, and TNF-α mRNA levels in rat primary microglial cells (Additional file 1: Figure S6a–f). In addition, pretreatment with CA140 significantly decreased the mRNA levels of IL-6 and iNOS in rat primary astrocytes (Additional file 1: Figure S6g–l).

**Fig. 3:**
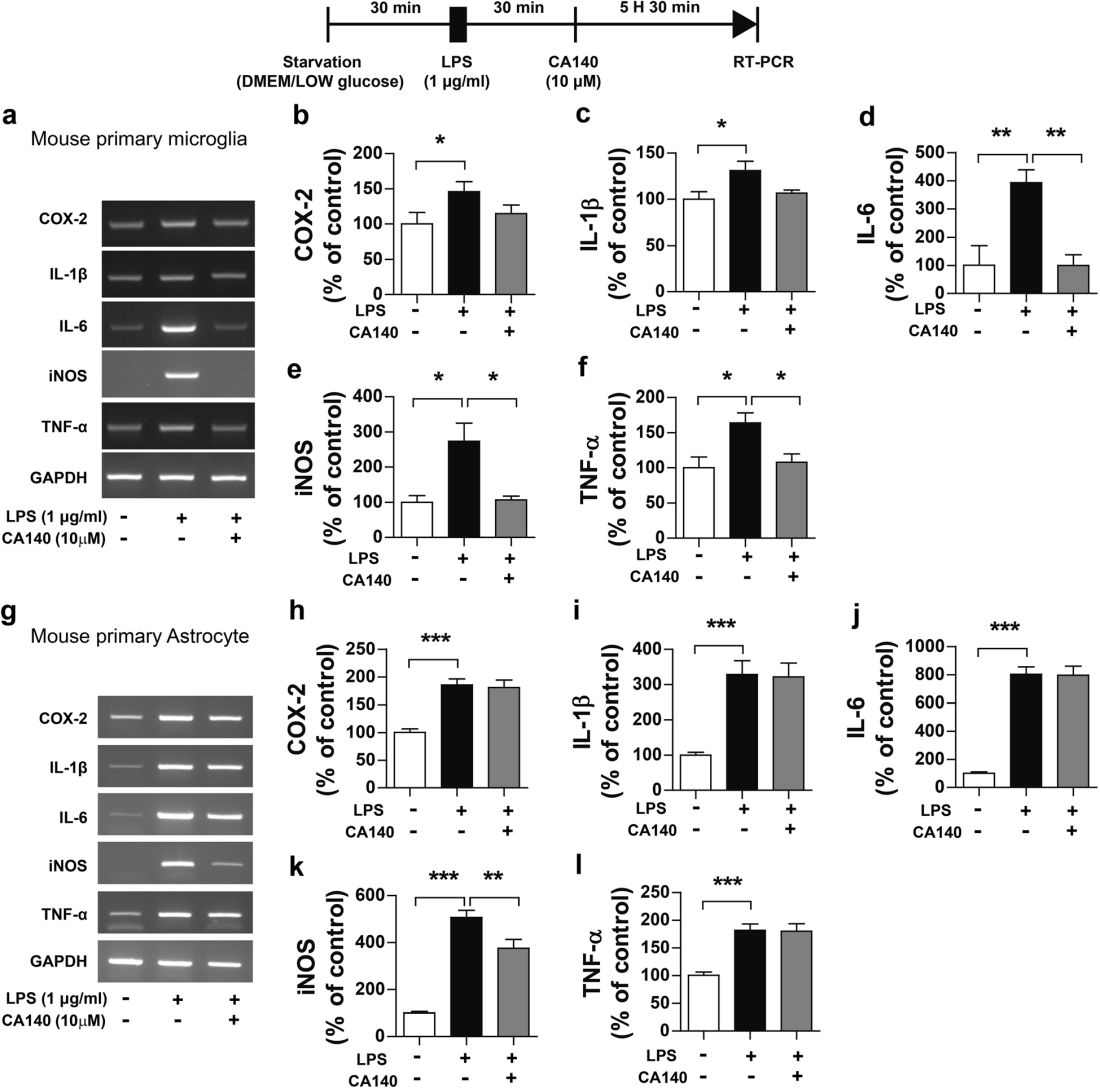
Pretreatment with LPS followed by CA140 treatment decreased LPS-induced proinflammatory cytokine levels in mouse primary microglial cells. **a–f** Mouse primary microglial cells were pretreated with LPS (1 μg/mL) or PBS for 30 min, followed by treatment with vehicle (1% DMSO) or CA140 (10 μM) for 5.5 h. Total RNA was then isolated, and proinflammatory cytokine levels were measured by RT-PCR (con, *n* = 4; LPS, *n* = 4; LPS + CA140, *n* = 4). **g–l** Mouse primary astrocytes were pretreated with LPS (1 μg/mL) or PBS for 30 min, followed by treatment with vehicle (1% DMSO) or CA140 (10 μM) for 5.5 h. Total RNA was then isolated, and proinflammatory cytokine levels were measured by RT-PCR (COX-2: con, *n* = 20; LPS, *n* = 20; LPS + CA140, *n* = 20; IL-1β: con, *n* = 20; LPS, *n* = 20; LPS + CA140, *n* = 20; IL-6, iNOS, and TNF-alpha: con, *n* = 16; LPS, *n* = 16; LPS + CA140, *n* = 16). *p < 0.05, **p < 0.001, ***p < 0.0001

Several recent studies have demonstrated that primary glial cells can be activated by high glucose levels [25–28]. Thus, we cultured primary glial cells under low-glucose DMEM conditions to test whether pre-or post-treatment with CA140 can differentially affect LPS-induced neuroinflammatory responses under low-glucose conditions. Mouse primary microglial cells were treated with LPS (1 μg/mL) or PBS for 30 min, followed by vehicle (1% DMSO) or CA140 (10 μM) for 5.5 h, and proinflammatory cytokine levels were measured by RT-PCR. Post-treatment with CA140 significantly reduced IL-6, iNOS, and TNF-alpha mRNA levels in LPS-stimulated mouse primary microglial cells (Fig. 3a–f). Interestingly, post-treatment with CA140 significantly suppressed LPS-stimulated iNOS mRNA levels in primary astrocytes but not the levels of other proinflammatory cytokines (Fig. 3g–l). In addition, pretreatment with CA140 significantly decreased LPS-induced proinflammatory cytokine levels in mouse primary microglial cells and primary astrocytes (Additional file 1: Figure S7a–l). These results indicate that the timing of CA140 treatment and culture conditions (high vs low glucose) can differentially affect the LPS-induced proinflammatory response depending on cell type. The alleviatory effect of CA140 was specific to microglial cells.

### CA140 regulates the dopamine D1 receptor to alter proinflammatory cytokine levels

Because CA140 is structurally related to DA, we hypothesized that CA140 may directly or indirectly interact with DA receptors (i.e., D1R, D2R) to alter the neuroinflammatory response. To test this idea, we initially investigated whether BV2 microglial cells present endogenous dopamine D1 receptor (D1R) or dopamine D2 receptor (D2R). For the initial experiment, BV2 microglial cells were pretreated with LPS (1 μg/mL) or PBS for 30 min, followed by treatment with CA140 (10 μM) or vehicle (1% DMSO) for 5.5 h, and the mRNA levels of D1R were measured by RT-PCR. Interestingly, LPS treatment significantly increased D1R mRNA levels, whereas treatment with LPS followed by CA140 significantly downregulated D1R mRNA levels (Additional file 1: Figure S8a–b). To further confirm these findings, we performed immunocytochemistry with anti-CD11b and anti-D1R antibodies and determined that post-treatment with CA140 reduced D1R levels compared with LPS treatment (Additional file 1: Figure S8c, d).

We subsequently examined whether post-treatment with CA140 can alter D2R levels. BV2 microglial cells were pretreated with LPS (1 μg/mL) or PBS for 30 min, followed by treatment with CA140 (10 μM) or vehicle (1% DMSO) for 5.5 h and immunocytochemistry with anti-CD11b and anti-D2R antibodies. Interestingly, LPS treatment significantly increased D2R levels in BV2 microglial cells (Additional file 1: Figure S8e, f). However, post-treatment with CA140 did not alter D2R levels compared with LPS treatment (Additional file 1: Figure S8e, f), which suggests that CA140 modulates only LPS-induced D1R expression levels in BV2 microglial cells.

**Fig. 4:**
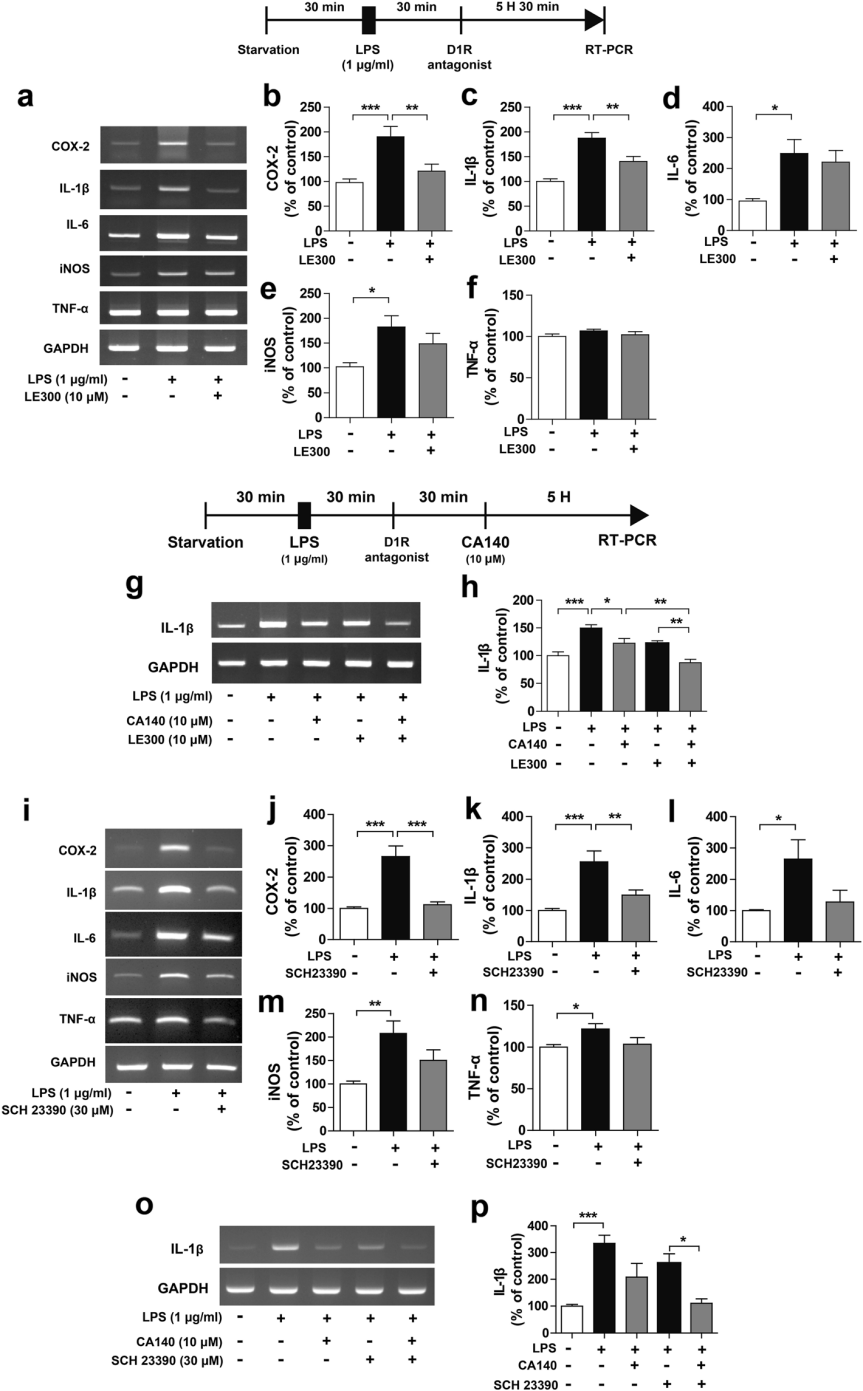
CA140 downregulated LPS-induced neuroinflammatory responses with D1R inhibition. **a** Representative images of proinflammatory cytokine mRNA levels in BV2 microglial cells. **b–f** BV2 microglial cells were pretreated with LPS (1 μg/mL) or PBS for 30 min, followed by treatment with vehicle (1% DMSO) or LE300 (a D1R antagonist, 10 μM) for 5.5 h 30 min. Total RNA was then isolated, and proinflammatory cytokine levels were measured by RT-PCR (IL-1β: con, *n* = 20; LPS, *n* = 20; LPS + CA140, *n* = 20; COX-2, IL-6, iNOS, and TNF-alpha: con, *n* = 10; LPS, *n* = 10; LPS + CA140, *n* = 10). **g–h** BV2 microglial cells were pretreated with LPS (1 μg/mL) or PBS for 30 min, followed by treatment with vehicle (1% DMSO) or LE300 (10 μM) for 30 min and finally CA140 (10 μM) or vehicle (1% DMSO) for 5 h. Total RNA was then isolated, and IL-1β mRNA levels were measured by RT-PCR (con, *n* = 16; LPS, *n* = 16; LPS + CA140, *n* = 16). **i–n** BV2 microglial cells were pretreated with LPS (1 μg/mL) or PBS for 30 min, followed by treatment with vehicle (1% DMSO) or SCH23390 (a D1R antagonist, 30 μM) for 5.5 h. Total RNA was then isolated, and proinflammatory cytokine levels were measured by RT-PCR (con, *n* = 12; LPS, *n* = 12; LPS + CA140, *n* = 12). **o–p** BV2 microglial cells were pretreated with LPS (1 μg/ml) or PBS for 30 min, followed by treatment with SCH23390 (30 μM) or vehicle (1% DMSO) for 30 min and finally CA140 (10 μM) or vehicle (1% DMSO) for 5 h. Total RNA was then isolated, and IL-1β mRNA levels were measured by RT-PCR (con, *n* = 14; LPS, *n* = 14; LPS + CA140, *n* = 14). *p < 0.05, **p < 0.001, ***p < 0.0001

To examine whether D1R or D2R affects the LPS-stimulated proinflammatory response, BV2 microglial cells were treated with LPS (1 μg/mL) or PBS for 30 min, followed by treatment with a D1R antagonist (LE300, 10 μM) or vehicle (1% DMSO) for 5.5 h. Proinflammatory cytokine levels were then measured by RT-PCR. LE300 treatment significantly reduced LPS-stimulated COX-2 and IL-1β mRNA levels (Fig. 4a–f), which suggests that inhibition of D1R regulates the proinflammatory response in LPS-induced BV2 microglial cells.

Next, to investigate whether CA140 further regulates the neuroinflammatory response in the presence of a D1R antagonist, BV2 microglial cells were pretreated with LPS (1 μg/mL) or PBS for 30 min, followed by treatment with LE300 (a D1R antagonist, 10 μM) or vehicle (1% DMSO) for 30 min and finally CA140 (10 μM) or vehicle (1% DMSO) for 5 h. Subsequent RT-PCR analysis revealed that treatment with LE300, CA140, and LPS further inhibited IL-1β mRNA levels compared with treatment with LE300 and LPS or CA140 and LPS (Fig. 4g–h).

To further confirm these findings, BV2 microglial cells were treated with LPS (1 μg/mL) or PBS for 30 min, followed by another D1R antagonist (SCH23390, 30 μM) or vehicle (1% DMSO) for 5.5 h, and proinflammatory cytokine levels were measured by RT-PCR. SCH23390 treatment significantly decreased LPS-induced COX-2 and IL-1β mRNA levels (Fig. 4i–n). In addition, treatment with SCH23390, CA140, and LPS further inhibited LPS-stimulated COX-2 and IL-1β mRNA levels compared with treatment with LPS and SCH23390 (Fig. 4o–p).

**Fig. 5:**
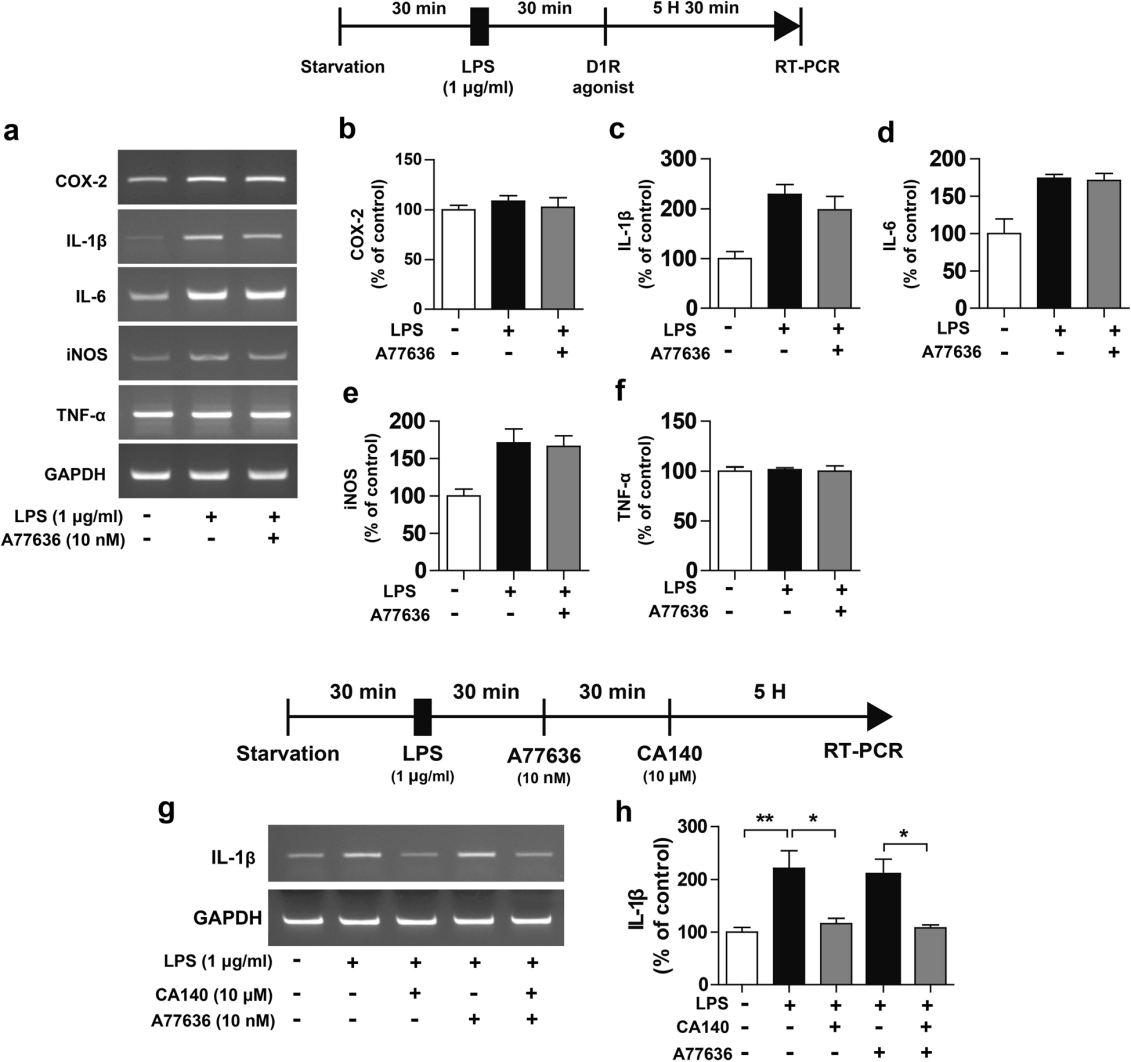
Post-treatment with CA140 significantly reduced LPS-induced IL-1β mRNA levels in the presence of a D1R agonist. **a** Representative images of proinflammatory cytokine mRNA levels in BV2 microglial cells. **b–f** BV2 microglial cells were pretreated with LPS (1 μg/mL) or PBS for 30 min, followed by treatment with vehicle (1% DMSO) or A77636 (a D1R agonist, 10 nM) for 5.5 h. Total RNA was then isolated, and proinflammatory cytokine levels were measured by RT-PCR (COX-2, IL-1β, IL-6, iNOS, and TNF-alpha: con, *n* = 4; LPS, *n* = 4; LPS + CA140, *n* = 4). **g–h** BV2 microglial cells were pretreated with LPS (1 μg/mL) or PBS for 30 min, followed by treatment with vehicle (1% DMSO) or A77636 (10 nM) for 30 min and finally CA140 (10 μM) or vehicle (1% DMSO) for 5 h. Total RNA was then isolated, and IL-1β mRNA levels were measured by RT-PCR (con, *n* = 6; LPS, *n* = 6; LPS + CA140, *n* = 6). *p < 0.05, **p < 0.001

Next, to examine whether D1R agonist treatment alters neuroinflammatory responses, BV2 microglial cells were treated with LPS (1 μg/mL) or PBS for 30 min, followed by a D1R agonist (A77636, 10 nM) or PBS for 5.5 h and measurement of proinflammatory cytokine levels by RT-PCR. A77636 treatment did not decrease any LPS-induced proinflammatory cytokine levels (Fig. 5a–f).

We then investigated whether CA140 modulates neuroinflammatory responses in the presence of a D1R agonist. BV2 microglial cells were pretreated with LPS (1 μg/mL) or PBS for 30 min, followed by treatment with A77636 (a D1R agonist, 10 nM) or PBS for 30 min and finally CA140 (10 μM) or vehicle (1% DMSO) for 5 h; we subsequently performed RT-PCR. Consistent with our findings above, post-treatment with CA140 dramatically reduced LPS-induced IL-1β mRNA levels (Fig. 5g–h). Most importantly, treatment with LPS, A77636, and CA140 significantly reduced LPS-induced IL-1β mRNA levels compared with treatment with A77636 and LPS (Fig. 5g–h).

We subsequently examined whether D2R inhibition affects the proinflammatory response in LPS-induced BV2 microglial cells and found that treatment with D2R antagonist did not suppress LPS-induced proinflammatory cytokine levels (Additional file 1: Figure S9a–f). In addition, treatment with LPS, EH, and CA140 did not decrease LPS-induced IL-1β mRNA levels compared with treatment with EH and LPS or CA140 and LPS (Additional file 1: Figure S9g–h). Taken together, these results suggest that CA140 may modulate D1R to alter proinflammatory responses.

### CA140 alters LPS-induced ERK signaling in BV2 microglial cells

**Fig. 6:**
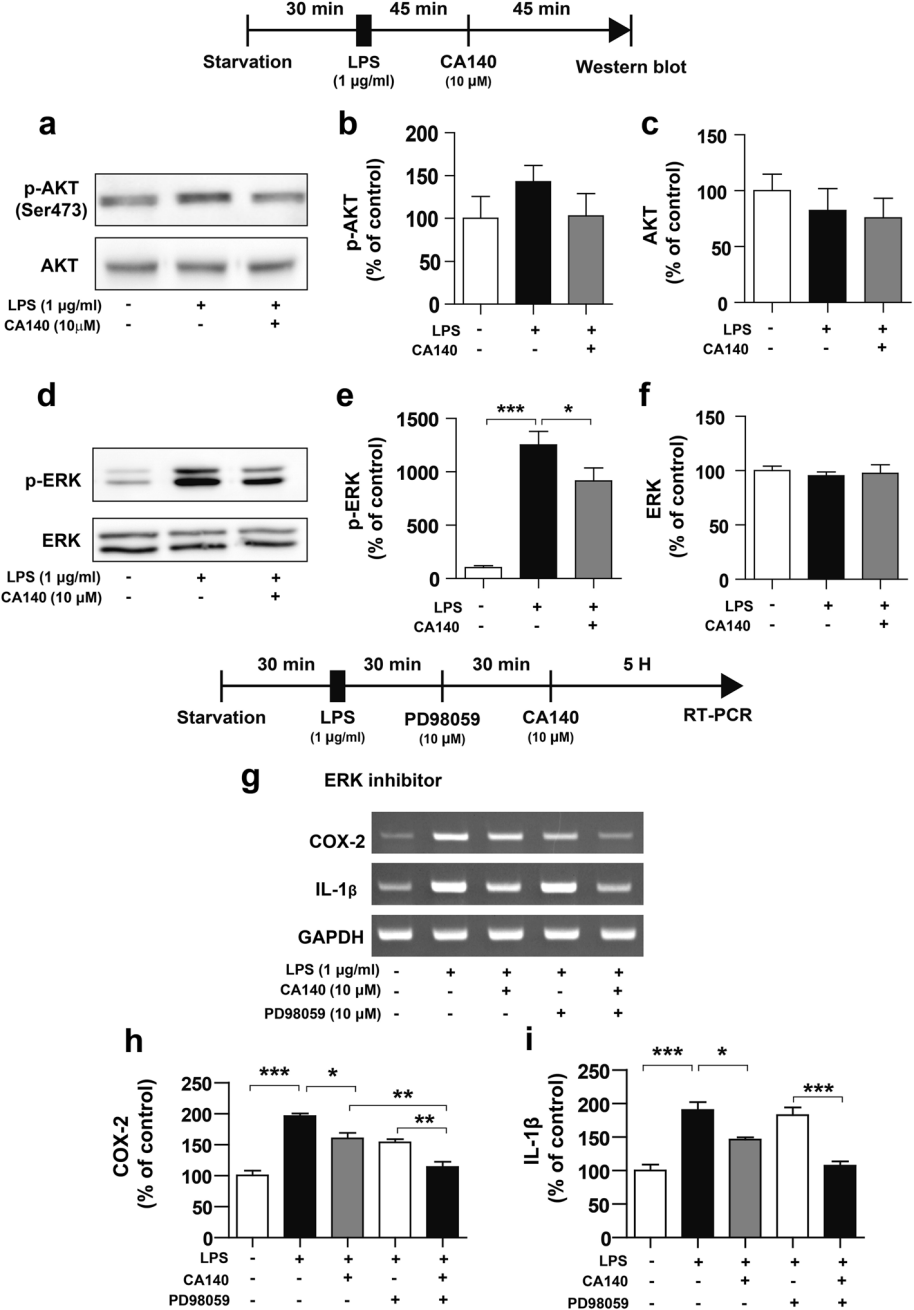
Pretreatment with LPS followed by CA140 treatment decreased ERK signaling in LPS-stimulated BV2 cells. **a–c** BV2 microglial cells were pretreated with LPS (1 μg/mL) or PBS for 45 min, followed by treatment with vehicle (1% DMSO) or CA140 (10 μM) for 45 min and western blotting with anti-p-AKT and anti-AKT antibodies (p-AKT and AKT; con, *n* = 5; LPS, *n* = 5; LPS + CA140, *n* = 5). **d–f** BV2 cells were pretreated with LPS (1 μg/ml) or PBS for 45 min, followed by treatment with vehicle (1% DMSO) or CA140 (10 μM) for 45 min and western blotting with anti-p-ERK and anti-ERK antibodies (p-ERK and ERK; con, *n* = 6; LPS, *n* = 6; LPS + CA140, *n* = 6). **g–i** BV2 microglial cells were pretreated with LPS (1 μg/mL) or PBS for 30 min, followed by treatment with an ERK inhibitor (PD98059, 10 μM) or vehicle (1% DMSO) for 30 min and finally CA140 (10 μM) or vehicle (1% DMSO) for 5 h. Total RNA was then isolated, and IL-1β or COX-2 mRNA levels were measured by RT-PCR (COX-2 and IL-1β: con, *n* = 4; LPS, *n* = 4; LPS + CA140, *n* = 4). *p < 0.05, **p < 0.001, ***p < 0.0001

Several studies have shown that ERK and AKT signaling plays an important role in regulating proinflammatory cytokines in microglial cells [29]. Thus, we investigated whether pre- or post-treatment with CA140 regulates ERK and AKT signaling to alter the LPS-induced neuroinflammatory response. BV2 microglial cells were pretreated with LPS (1 μg/mL) or PBS for 45 min, followed by treatment with CA140 (10 μM) or vehicle (1% DMSO) for 45 min and western blotting with anti-p-ERK/ERK or anti-p-AKT/AKT antibodies. Post-treatment with CA140 did not significantly alter p-AKT levels (Fig. 6a–c), whereas post-treatment with CA140 significantly decreased p-ERK levels in LPS-stimulated BV2 microglial cells (Fig. 6d–f).

We subsequently assessed whether post-treatment with CA140 regulates the LPS-stimulated proinflammatory response through ERK signaling. BV2 microglial cells were pretreated with LPS (1 μg/mL) or PBS for 30 min, followed by treatment with PD98059 (an ERK inhibitor, 10 μM) or vehicle (1% DMSO) for 30 min and finally vehicle (1% DMSO) or CA140 (10 μM) for 5 h. The mRNA levels of COX-2 and IL-1β were measured by RT-PCR. Consistent with our previously described findings, post-treatment with CA140 significantly decreased the mRNA levels of COX-2 and IL-1β (Fig. 6g–i). In addition, compared with LPS and PD98059 treatment, treatment with LPS, PD98059, and CA140 further decreased COX-2 and IL-1β mRNA levels (Fig. 6g–i).

We then examined whether pretreatment with CA140 alters ERK and AKT signaling. BV2 cells were pretreated with vehicle (1% DMSO) or CA140 (10 μM) for 45 min, followed by LPS (1 μg/mL) or PBS treatment for 45 min and western blotting with anti-p-ERK/ERK or anti-p-AKT/AKT antibodies. Interestingly, pretreatment with CA140 significantly suppressed the phosphorylation of ERK and AKT in LPS-treated BV2 microglial cells (Additional file 1: Figure S10a–f). These data suggest that pre- or post-treatment with CA140 differentially affects ERK and AKT signaling.

### CA140 suppresses LPS-induced cytosolic and nuclear p-STAT3 in BV2 microglial cells

**Fig. 7:**
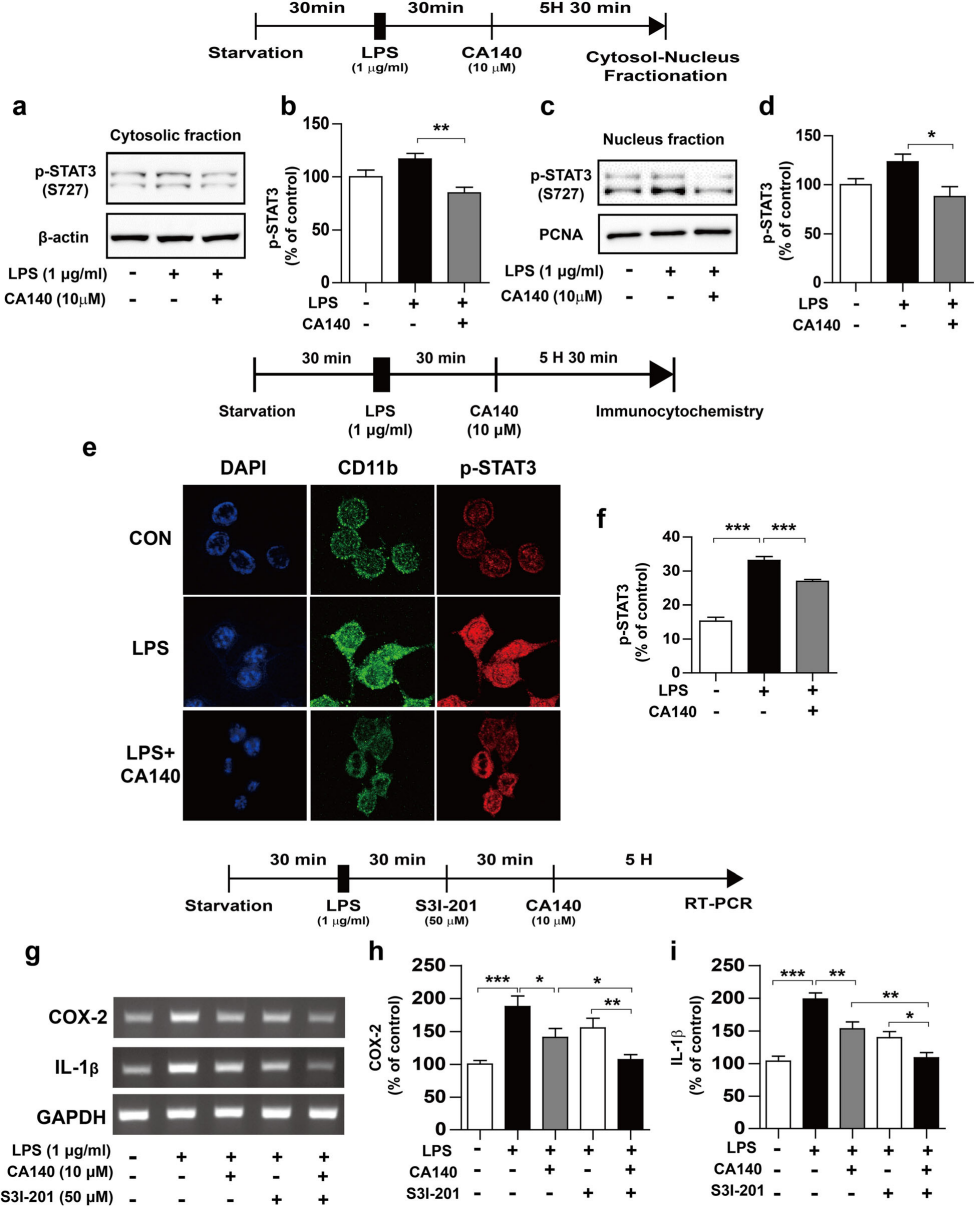
Pretreatment with LPS followed by CA140 treatment decreased phosphorylation of STAT3 in the nucleus and cytosol. **a** BV2 microglial cells were pretreated with LPS (1 μg/mL) or PBS for 45 min, followed by treatment with vehicle (1% DMSO) or CA140 (10 μM) for 5.5 h and subcellular fractionation (nuclear and cytosolic fractions). Western blotting was performed on the cytosolic fraction using antibodies against p-STAT3 (Ser727) and β-actin. **b** Quantification of data from **a** (con, *n* = 12; LPS, *n* = 12; LPS + CA140, *n* = 12). **c**, **d** Western blotting was performed on the nuclear fraction using anti-p-STAT3 (Ser727) and anti-PCNA antibodies (con, *n* = 12; LPS, *n* = 12; LPS + CA140, *n* = 12). **e**, **f** BV2 microglial cells were pretreated with LPS (1 μg/ml) or PBS for 30 min, followed by treatment with vehicle (1% DMSO) or CA140 (10 μM) for 5.5 h and immunostaining with anti-p-STAT3 (Ser727) and anti-CD11b antibodies (con, *n* = 202; LPS, *n* = 169; LPS + CA140, *n* = 397). **g–i** BV2 microglial cells were pretreated with LPS (1 μg/mL) or PBS for 30 min, followed by treatment with a STAT3 inhibitor (S3I-301, 10 μM) or vehicle (1% DMSO) for 30 min and finally CA140 (10 μM) or vehicle (1% DMSO) for 5 h. Total RNA was then isolated, and IL-1β or COX-2 mRNA levels were measured by RT-PCR (COX-2 and IL-1β: con, *n* = 17; LPS, *n* = 17; LPS + CA140, *n* = 17). *p < 0.05, **p < 0.01, ***p < 0.0001

STAT3 plays an important role in the regulation of proinflammatory cytokine levels induced by LPS [30]. Thus, we examined whether CA140 regulates STAT3 expression in the nucleus and cytosol. BV2 microglial cells were pretreated with LPS (1 μg/mL) or PBS for 30 min, followed by treatment with vehicle (1% DMSO) or CA140 (10 μM) for 5.5 h and subcellular fractionation. LPS treatment significantly increased p-STAT3 (Ser727) levels in the cytosol and nucleus (Fig. 7a–d). In addition, post-treatment with CA140 significantly reduced LPS-induced cytosolic and nuclear p-STAT3 (Ser727) levels (Fig. 7a–d). As a complementary study, we conducted immunocytochemistry with anti-CD11b and anti-p-STAT3 (Ser727) antibodies and determined that post-treatment with CA140 significantly decreased LPS-induced p-STAT3 (Ser727) levels in the nucleus (Fig. 7e–f).

In addition, we investigated whether pretreatment with CA140 alters LPS-stimulated p-STAT3 levels in the cytosol and nucleus. For this experiment, BV2 microglial cells were pretreated with vehicle (1% DMSO) or CA140 (10 μM) for 30 min, followed by treatment with LPS (1 μg/mL) or PBS for 5.5 h and subcellular fractionation. Pretreatment with CA140 also significantly decreased LPS-induced cytosolic and nuclear p-STAT3 (Ser727) levels (Additional file 1: Figure S11a–d).

We then examined whether CA140 further regulates the LPS-induced proinflammatory response in the presence of a STAT3 inhibitor. BV2 microglial cells were pretreated with LPS (1 μg/mL) or PBS for 30 min, followed by treatment with S31–201 (a STAT3 inhibitor, 50 μM) or vehicle (1% DMSO) for 30 min and finally vehicle (1% DMSO) or CA140 (10 μM) for 5 h. The mRNA levels of COX-2 and IL-1β were then measured by RT-PCR. Compared with treatment with LPS and S3I-201 or with LPS and CA140, treatment with LPS, S3I-201, and CA140 further decreased IL-1β and COX-2 mRNA levels (Fig. 7g–i). These data suggest that CA140 modulates STAT3 signaling to regulate the LPS-stimulated neuroinflammatory response.

### CA140 significantly reduces the activation of microglia and astrocytes in LPS-injected wild-type mice

**Fig. 8:**
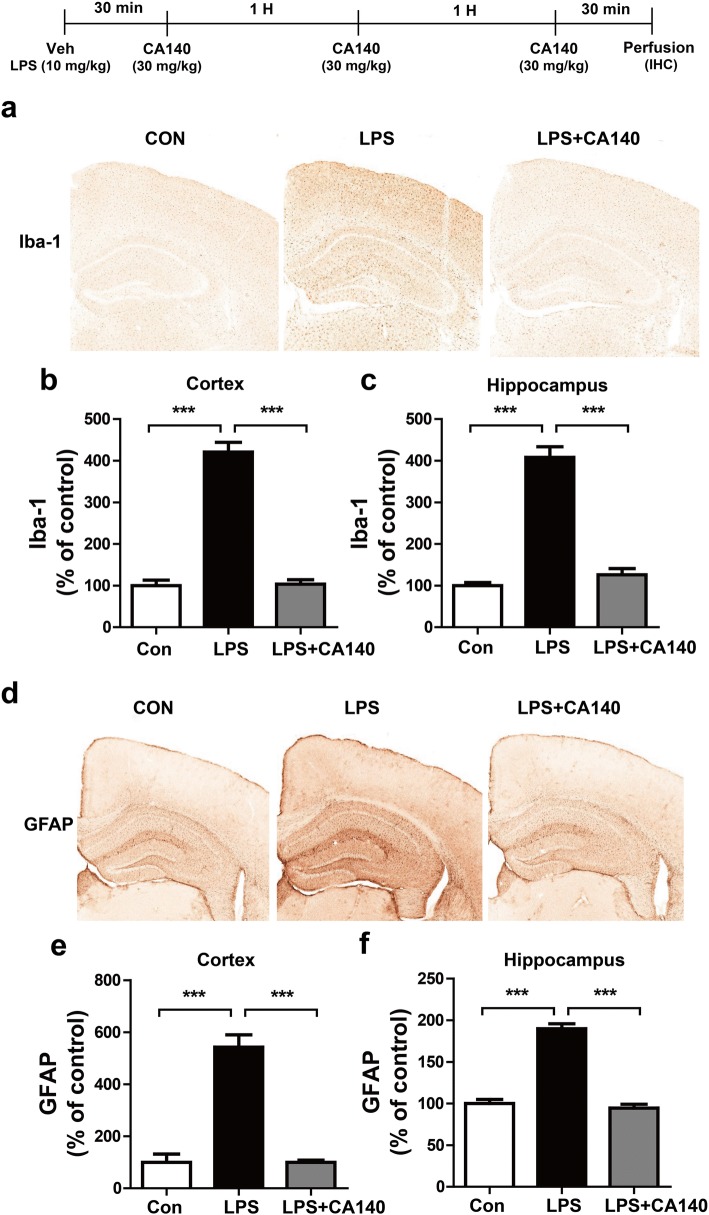
Pretreatment with LPS followed by CA140 treatment significantly decreased microglial and astrocyte activation in wild-type mice. **a** Wild-type mice were injected with LPS (10 mg/kg, i.p.), followed 30 min later by injection twice with CA140 (30 mg/kg, i.p.) at an interval of 1 h and a third injection (30 mg/kg, i.p.) at an interval of 30 min. The mice were perfused, fixed, and immunostained with anti-Iba-1 antibody. **b**, **c** Quantification of data from **a** (con, *n* = 5 mice; LPS, *n* = 5 mice; LPS + CA140, *n* = 5 mice). **d** Wild-type mice were injected with LPS (10 mg/kg, i.p.), followed 30 min later by injection twice with CA140 (30 mg/kg, i.p.) at an interval of 1 h and a third injection (30 mg/kg, i.p.) at an interval of 30 min. The mice were perfused, fixed, and immunostained with anti-GFAP antibody. **e**, **f** Quantification of data from **d** (con, *n* = 5 mice; LPS, *n* = 5 mice; LPS + CA140, *n* = 5 mice). ***p < 0.0001

Numerous studies have shown that activated microglia and astrocytes are involved in neuroinflammatory responses [31]. To examine whether post-treatment with CA140 alters microglial and astrocyte activation in vivo, wild-type mice were injected with LPS (10 mg/kg/day, i.p.) or PBS, followed 30 min later by injection with CA140 (30 mg/kg, i.p., twice with an interval of 1 h, followed 30 min later by a third injection) or vehicle (10% DMSO), and immunohistochemistry was performed with anti-Iba-1 and anti-GFAP antibodies. As expected, LPS-injected wild-type mice exhibited significantly increased Iba-1 (Fig. 8a–c) and GFAP (Fig. 8d–f) immunoreactivity in the hippocampus and cortex. In addition, post-treatment with CA140 significantly reduced microglial (Fig. 8a–c) and astrocyte (Fig. 8d–f) immunoreactivity compared with LPS-injected wild-type mice.

**Fig. 9:**
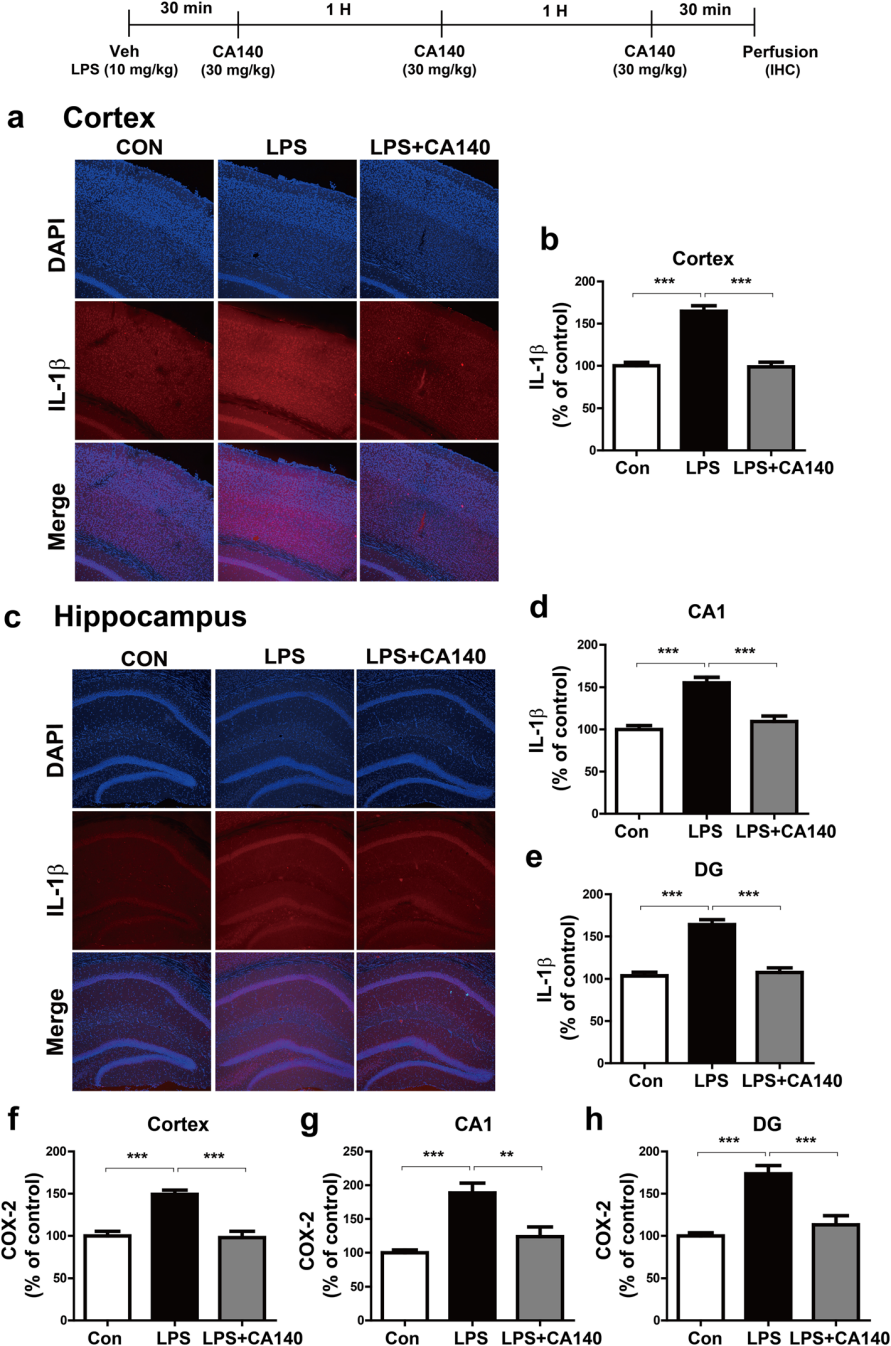
Pretreatment with LPS followed by CA140 treatment significantly reduced IL-1β and COX-2 levels in wild-type mice. **a**, **c** Wild-type mice were injected with LPS (10 mg/kg, i.p.), followed 30 min later by injection twice with CA140 (30 mg/kg, i.p.) at an interval of 1 h and a third injection (30 mg/kg, i.p.) at an interval of 30 min. The mice were perfused, fixed, and immunostained with anti-IL-1β antibody in the cortex (**a**) and hippocampus (**c**). **b**, **d**, **e** Quantification of data from **a** (cortex: con, *n* = 5 mice; LPS, *n* = 5 mice; LPS + CA140, *n* = 5 mice) and **c** (hippocampus CA1 and DG: con, *n* = 5 mice; LPS, *n* = 5 mice; LPS + CA140, *n* = 5 mice). **f**–**h** Wild-type mice were injected with LPS (10 mg/kg, i.p.), followed 30 min later by injection twice with CA140 (30 mg/kg, i.p.) at an interval of 1 h and a third injection (30 mg/kg, i.p.) at an interval of 30 min. The mice were perfused, fixed, and immunostained with anti-IL-1β antibody in the cortex (con, *n* = 5 mice; LPS, *n* = 5 mice; LPS + CA140, *n* = 5 mice), hippocampus CA1 (**g**, con, *n* = 5 mice; LPS, *n* = 5 mice; LPS + CA140, *n* = 5 mice), and dentate gyrus (**h**, con, *n* = 5 mice; LPS, *n* = 5 mice; LPS + CA140, *n* = 5 mice). ***p < 0.0001

We subsequently examined whether post-treatment with CA140 alters LPS-stimulated proinflammatory cytokine levels in wild-type mice. For this experiment, wild-type mice were injected with LPS (10 mg/kg/day, i.p.) or PBS, followed 30 min later by injection with CA140 (30 mg/kg, i.p., twice with an interval of 1 h, followed 30 min later by a third injection) or vehicle (10% DMSO, i.p). Immunohistochemistry was performed with anti-IL-1β and anti-COX-2 antibodies. Post-treatment with CA140 significantly downregulated LPS-stimulated IL-1β (Fig. 9a–e) and COX-2 (Fig. 9f–h) immunoreactivity in the cortex and hippocampus.

To further examine whether pretreatment with CA140 attenuates microglial and astrocyte activation, wild-type mice were injected with CA140 (30 mg/kg, i.p.) or vehicle (10% DMSO, i.p.) daily for 3 days, followed by injection with LPS (10 mg/kg/day, i.p.) or PBS. Three hours later, immunohistochemistry was conducted with anti-Iba-1 and anti-GFAP antibodies. We observed that pretreatment with CA140 also significantly decreased microglial and astrocyte immunoreactivity (Additional file 1: Figure S12a–f). These results suggest that CA140 may be beneficial for the prevention and treatment of neuroinflammatory-related disease.

### CA140 significantly decreases the activation of microglia and astrocytes in a mouse model of AD

**Fig. 10:**
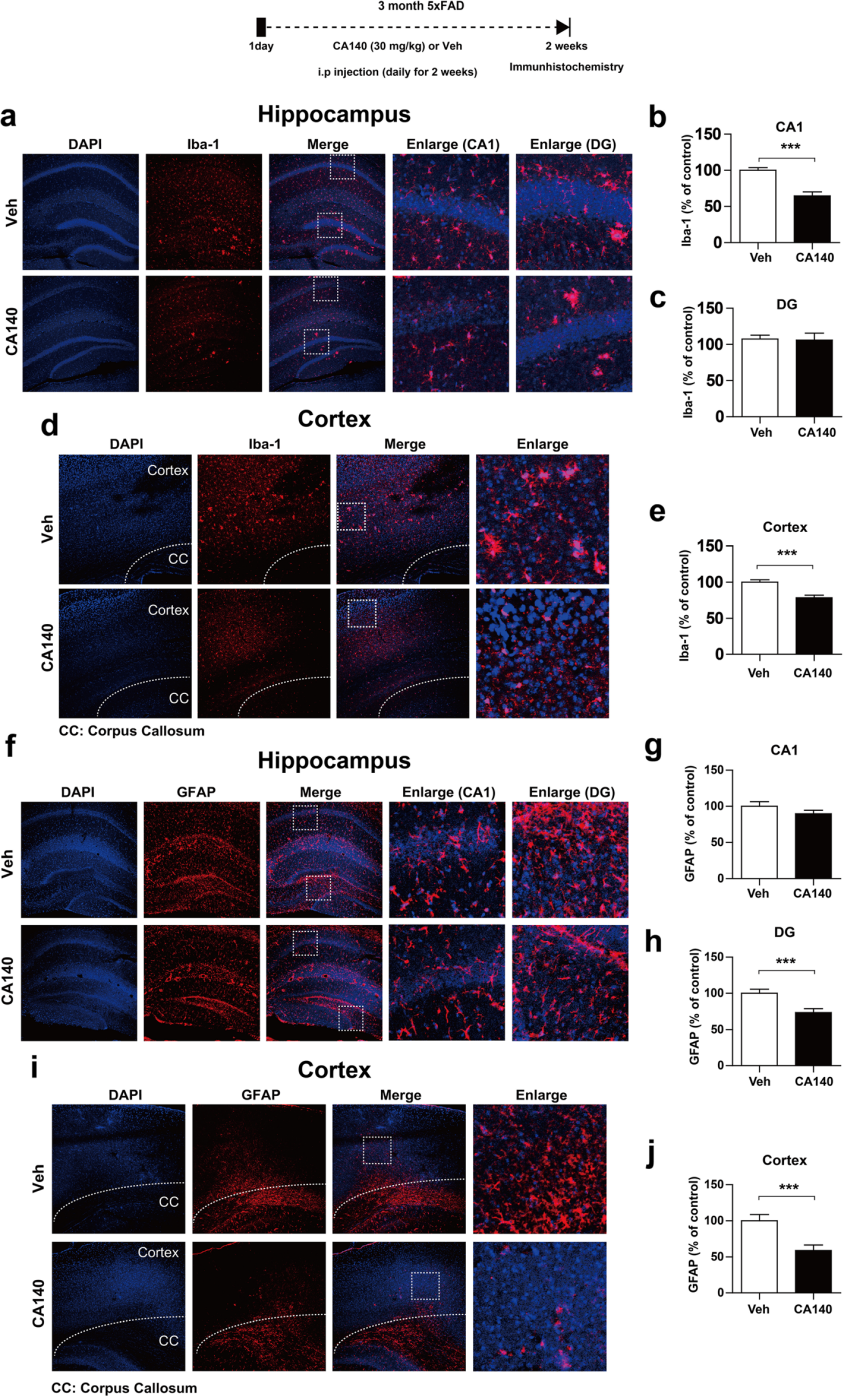
CA140 significantly reduced microglial and astrocyte activation in 5xFAD mice. **a**, **d** Representative images of the hippocampus (**a**) and cortex (**d**) from 5xFAD mice injected with vehicle (10% DMSO, i.p.) or CA140 (30 mg/kg, i.p.) daily for 2 weeks; immunohistochemistry was performed with anti-Iba-1 antibody. **b**, **c** Quantification of data from **a** (hippocampus CA1 and DG: con, *n* = 8 mice; CA140, *n* = 8 mice). **e** Quantification of data from **d** (cortex: con, *n* = 8 mice; CA140, *n* = 8 mice). **f**, **i** Representative images of the hippocampus (**f**) and cortex (**i**) from 5xFAD mice injected with vehicle (10% DMSO, i.p.) or CA140 (30 mg/kg, i.p.) daily for 2 weeks; immunohistochemistry was performed with anti-GFAP antibody. **g**, **h** Quantification of data from **f** (hippocampus CA1 and DG: con, *n* = 6 mice; CA140, *n* = 6 mice). **j** Quantification of data from **i** (cortex: con, *n* = 6 mice; CA140, *n* = 6 mice). ***p < 0.0001

Neuroinflammation and microglial activation are closely associated with neurodegenerative diseases, including AD [32–34]. Thus, we aimed to examine whether CA140 regulates microglial and astrocyte activation in a mouse model of AD. 5xFAD mice (3 months old) were injected with CA140 (30 mg/kg, i.p.) or vehicle (10% DMSO, i.p.) daily for 2 weeks. After 2 weeks, immunohistochemistry was performed with anti-Iba-1 (a microglial cell marker, Fig. 10a–e) and anti-GFAP (an astrocyte marker, Fig. 10f–j) antibodies. CA140-injected 5xFAD mice had significantly reduced Iba-1 immunoreactivity in the hippocampus CA1 (Fig. 10a, b) and cortex (Fig. 10d, e) but not the dentate gyrus (Fig. 10a, c). Furthermore, CA140-injected 5xFAD mice had significantly suppressed GFAP immunoreactivity in the hippocampus DG (Fig. 10f, h) and cortex (Fig. 10i, j) but not the hippocampus CA1 (Fig. 10f, g). These data suggest that CA140 modulates microglial and astrocyte activation in a mouse model of AD.

## Discussion

Increasing evidence is highlighting the critical role of the immune system in neurodegenerative diseases such as AD. Unchecked glial activation and neuroinflammation may represent hallmark diagnostic features of neurodegenerative diseases. However, research to explicate the mechanisms underlying neuroinflammation has been limited.

Several recent studies have demonstrated that microglia and astrocytes release proinflammatory cytokines, leading to neuronal cell death and synaptic dysfunction in neurodegenerative diseases, including AD [35, 36]. The release of these cytokines may be induced by LPS in vivo and in vitro via Toll-like receptors [37, 38]. McGeer et al. determined that neuroinflammation stimulated by a single intraperitoneal injection of LPS lasted 10 months in the mouse brain and eventually led to neurodegeneration. Therefore, the identification of agents that reduce proinflammatory cytokine levels may represent a promising strategy for developing drugs to treat neurodegenerative diseases.

In this study, we synthesized a novel analog of dopamine, CA140 that can penetrate the blood-brain barrier. We determined that 10 μM CA140 was effective for lowering LPS-induced proinflammatory cytokine levels in BV2 microglial cells regardless of the timing of treatment (Fig. 2, Additional file 1: Figure S1). However, post-treatment with 5 μM CA140 only reduced the mRNA levels of LPS-induced IL-1β and not those of other proinflammatory cytokines. Our findings imply that an appropriate concentration of CA140 may be efficiently employed to both reduce and prevent neuroinflammatory responses in LPS-stimulated BV2 microglial cells. In addition, we observed that pretreatment with CA140 significantly reduced LPS-induced proinflammatory cytokine levels in rat primary microglia and primary astrocytes under high-glucose conditions (Additional file 1: Figure S6). However, post-treatment with CA140 only affected the LPS-stimulated proinflammatory response in rat primary microglial cells and not primary astrocytes under high-glucose conditions (Additional file 1: Figure S6). Why do pre- and post-treatment with CA140 have different effects on LPS-induced proinflammatory responses? Several recent studies have reported that high glucose levels induce primary glial cell activation [25–28]. Thus, we conducted additional experiments to assess the anti-inflammatory effects of CA140 on primary glial cells under low-glucose conditions. Pre- or post-treatment with CA140 significantly reduced LPS-stimulated proinflammatory cytokine levels in mouse primary microglial cells under low-glucose conditions (Fig. 3a–f, Additional file 1: Figure S7). In addition, pretreatment with CA140 significantly reduced LPS-induced proinflammatory cytokine levels in mouse primary astrocytes (Additional file 1: Figure S7). Interestingly, post-treatment with CA140 only reduced LPS-induced iNOS mRNA levels in mouse primary astrocytes under low-glucose conditions (Fig. 3). These data suggest that pre- or post-treatment with CA140 may have different effects depending on cell type and culture conditions (e.g., low vs high glucose).

The physiological functions of the catecholaminergic neurotransmitter DA, which range from voluntary movement and reward to hormonal regulation and hypertension, are mediated by G-protein-coupled DA receptors (D1, D2, D3, D4, and D5) [39, 40]. DA receptors have also been identified as important factors for controlling immunity in the CNS [41]. Importantly, D1R and D2R are expressed in rodent and human microglia from brains damaged by stroke or neurodegeneration [41–43]. Here, we observed that D1R and D2R were expressed in BV2 microglial cells and upregulated by LPS treatment (Additional file 1: Figure S8). Previous studies and our results may imply that the upregulation of DA receptors in microglia contributes to neuroinflammation in pathological conditions. Spiperone, a D1/D2R antagonist, inhibits DA-induced chemotaxis in cultured human microglia [44]. Pretreatment with SCH23390, an antagonist of D1R, suppresses NO production by microglia in LPS-injected mice [45]. Consistent with these findings, pretreatment with LE300 or SCH23390, antagonists of D1R, significantly suppressed COX-2 and IL-1β mRNA levels in LPS-stimulated BV2 microglial cells (Fig. 4). A68930, an agonist of D1R, inhibits the production of proinflammatory cytokines in mice [46], and pretreatment with SKF83959, an atypical D1R agonist, reduces proinflammatory cytokine levels in LPS-stimulated BV2 microglia [47]. However, in the present study, pretreatment with A77636, a selective agonist of D1R, did not reduce LPS-induced proinflammatory cytokine levels (Fig. 5). More importantly, treatment with A77636, LPS, and CA140 significantly suppressed LPS-stimulated IL-1β mRNA levels compared with treatment with A77636 and LPS, suggesting that CA140 regulates D1R to alter the LPS-induced neuroinflammatory response (Fig. 5).

With respect to the effects of D2R on neuroinflammation, the D2R agonist pramipexole increases nitrites in cultured primary microglia [42]. In addition, sulpiride, an antagonist of D2R, reduces LPS-induced TNF-α and NO production [45]. In astrocytes, D2R contributes to the suppression of neuroinflammation; however, microglial D2R is not involved in neuroinflammation according to studies of D2R-deficient mice or an ischemic mouse model [48, 49]. In our study, the D2R antagonist eticlopride hydrochloride (EH) did not alter LPS-stimulated proinflammatory cytokine levels in BV2 microglial cells (Additional file 1: Figure S9). This discrepancy may be a result of differences in the details of the experimental procedures, such as treatment duration (i.e., 6 h compared with 24 h), the effective dose of antagonist or agonist, and/or pre- or post-treatment with a DA receptor antagonist. Based on the existing literature and our current findings, we suggest that CA140 may directly or indirectly interact with D1R and thereby regulate neuroinflammatory responses. However, we do not exclude other possibilities; for example, CA140 may regulate other neuroinflammation-related receptors (e.g., TLR4, other DA receptors) to modulate neuroinflammatory responses. Additional studies are required to fully dissect the molecular mechanisms involved in the CA140/DA receptors-induced neuroinflammatory response in vivo.

Activation of TLR receptors via LPS turns on downstream signaling cascades, such as MAP kinases, including ERK and AKT signaling in microglia and astrocytes [10, 50, 51]. Therefore, inhibiting the MAP kinase signaling pathway has been suggested as a potential target for therapeutic drugs for anti-inflammation. Moreover, MAP kinase has been suggested as a downstream effector of both D1R and D2R stimulation [52, 53]. Treatment with the D1R agonist SKF 38393 and the D2R agonist quinpirole activates ERK signaling in primary cultured striatal neurons [54], and in cultured neuroblastoma cells, treatment with the D1R agonist SKF 38393 results in oxidative stress and cytotoxicity via ERK activation [55]. Interestingly, our results indicated that pre- or post-treatment with CA140 significantly suppressed LPS-stimulated ERK signaling in BV2 microglial cells (Fig. 6, Additional file 1: Figure S10). In addition, we found that CA140 further reduced proinflammatory cytokine levels when combined with an ERK inhibitor, which suggests that CA140 alters LPS-induced ERK phosphorylation to modify the neuroinflammatory response.

STAT3, a member of the STAT family, is a transcription factor that plays a critical role in regulating microglial activation and inflammatory responses [56, 57]. STAT3 levels in microglia are enhanced in brain injury and a neurodegenerative disease model [58, 59]. Thus, we examined whether CA140 alters the nuclear localization of STAT3 to regulate the neuroinflammatory response and found that pre- or post-treatment with CA140 reduced cytosolic and nuclear p-STAT3 levels in LPS-stimulated BV2 microglial cells (Fig. 7, Additional file 1: Figure S11). Taken together, our data suggest that CA140 may alter neuroinflammation by regulating the ERK/STAT3 signaling pathway.

Systemic injection of LPS in wild-type mice significantly induces astrocyte and microglial activation and proinflammatory cytokine expression [60–62]. Moreover, a single injection of LPS induces robust expression of IL-1β and TNF-α mRNA in various brain regions of wild-type mice [63]. Intracerebral LPS injection in rats induces inflammatory responses and β-secretase-1 (BACE1) in the cortex and hippocampus, with axonal and dendritic pathologies similar to those present in AD [64, 65]. Other studies have also demonstrated that LPS treatment exacerbates the accumulation of amyloid beta and tau pathology in a mouse model of AD [59, 66, 67]. Interestingly, a recent study has shown that both SCH23390, a D1R antagonist, and sulpiride, a D2R antagonist, suppress proinflammatory cytokine levels in LPS-injected mice [45]. DA released by electroacupuncture reduces proinflammatory cytokine levels through D1R in LPS-injected mice [68]. In addition, the regulation of catecholamines by pharmacological agents, such as methylphenidate, enhances neuroinflammatory responses and microgliosis in 5xFAD mice, a transgenic AD mouse model [32, 69]. In the present study, our novel drug CA140, which is structurally related to DA, also substantially reduced astrocyte and microglial activation as well as proinflammatory cytokine levels in LPS-injected wild-type mice and 5xFAD mice (Fig. 8–10, Additional file 1: Figure S12). Taken together, our results suggest that CA140 may serve as a therapeutic agent for the prevention/treatment of neuroinflammation-related diseases, including AD.

## Conclusions

In the present study, we have demonstrated that CA140 exhibits novel anti-inflammatory effects and provided initial evidence for its mechanism of action. We discovered that CA140 suppresses LPS-induced proinflammatory cytokine levels in BV2 microglial cells, primary microglia, and primary astrocytes. In addition, CA140 modulates D1R to regulate LPS-induced neuroinflammation in BV2 microglial cells. Moreover, CA140 affects ERK/STAT3 signaling to alter the LPS-induced neuroinflammatory response. In in vivo experiments, CA140 significantly reduced microglial and astrocyte activation in LPS-injected wild-type mice and 5xFAD mice. This study reveals that CA140 is a promising compound with anti-inflammatory effects and subsequent attenuation of neurotoxins such as LPS.

## Additional file

Additional file 1: **Figure S1.** Post-treatment with CA140 at 5 μM only significantly reduced LPS-induced IL-1β mRNA levels. Figure S2. Post-treatment with CA140 significantly reduced LPS-induced proinflammatory cytokine levels in a longer treatment. Figure S3. Pretreatment with CA140 significantly decreased LPS-induced COX-2, IL-1β, and iNOS mRNA levels in BV2 microglial cells. Figure S4. Pretreatment with CA140 significantly decreased LPS-induced COX-2, IL-1β, and iNOS mRNA levels in a longer treatment. Figure S5. Post-treatment with CA140 significantly decreased LPS-mediated proinflammatory cytokine levels in rat primary microglial cells. Figure S6. Pretreatment with CA140 decreased LPS-induced proinflammatory cytokine levels in rat primary microglial cells and primary astrocytes. Figure S7. Pretreatment with CA140 decreased LPS-mediated proinflammatory cytokine levels in mouse primary microglial cells and primary astrocytes. Figure S8. Post-treatment with CA140 downregulated LPS-induced dopamine D1 receptor (D1R) levels in BV2 microglial cells. Figure S9. Inhibition of dopamine D2 receptor (D2R) did not reduce LPS-stimulated proinflammatory cytokine levels in BV2 microglial cells. Figure S10 Pretreatment with CA140 significantly decreased phosphorylation of ERK and AKT in LPS-stimulated BV2 microglial cells. Figure S11. Pretreatment with CA140 significantly decreased cytosolic and nuclear p-STAT3 levels in LPS-induced BV2 microglial cells. Figure S12. Pretreatment with CA140 significantly reduced microglia and astrocyte activation in wild-type mice. (DOCX 22915 kb)

## Abbreviations

AD: Alzheimer’s disease
BBB: Blood-brain barrier
CNS: Central nervous system
D1R: Dopamine D1 receptor
D2R: Dopamine D2 receptor
DA: Dopamine
ERK: Extracellular signal-regulated kinase
IL-1β: Interleukin-1β
JNK: c-Jun N-terminal kinase
LPS: Lipopolysaccharide
MAPK: Mitogen-activated protein kinase
TLR4: Toll-like receptor 4
TNF-α: Tumor necrosis factor-α
