# Supplementary material for: The small molecule CA140 inhibits the neuroinflammatory response in wild-type mice and a mouse model of AD
Source: J Neuroinflammation. 2018 Oct 11;15:286. doi: 10.1186/s12974-018-1321-3 (PMC6182807; doi:10.1186/s12974-018-1321-3)
Supplement: Supplementary file 1 — Figure S1. Post-treatment with CA140 at 5 μM only significantly reduced LPS-induced IL-1β mRNA levels. Figure S2. Post-treatment with CA140 significantly reduced LPS-induced proinflammatory cytokine levels in a longer treatment. Figure S3. Pretreatment with CA140 significantly decreased LPS-induced COX-2, IL-1β, and iNOS mRNA levels in BV2 microglial cells. Figure S4. Pretreatment with CA140 significantly decreased LPS-induced COX-2, IL-1β, and iNOS mRNA levels in a longer treatment. Figure S5. Post-treatment with CA140 significantly decreased LPS-mediated proinflammatory cytokine levels in rat primary microglial cells. Figure S6. Pretreatment with CA140 decreased LPS-induced proinflammatory cytokine levels in rat primary microglial cells and primary astrocytes. Figure S7. Pretreatment with CA140 decreased LPS-mediated proinflammatory cytokine levels in mouse primary microglial cells and primary astrocytes. Figure S8. Post-treatment with CA140 downregulated LPS-induced dopamine D1 receptor (D1R) levels in BV2 microglial cells. Figure S9. Inhibition of dopamine D2 receptor (D2R) did not reduce LPS-stimulated proinflammatory cytokine levels in BV2 microglial cells. Figure S10 Pretreatment with CA140 significantly decreased phosphorylation of ERK and AKT in LPS-stimulated BV2 microglial cells. Figure S11. Pretreatment with CA140 significantly decreased cytosolic and nuclear p-STAT3 levels in LPS-induced BV2 microglial cells. Figure S12. Pretreatment with CA140 significantly reduced microglia and astrocyte activation in wild-type mice. (DOCX 22915 kb) [file 12974_2018_1321_MOESM1_ESM.docx]

**Supplementary Materials**

**Small molecule CA140 inhibits the neuroinflammatory response in wild-type mice and a mouse model of AD**

Ju-Young Lee^1^, Jin Han Nam^1^, Youngpyo Nam^1,^ Hye Yeon Nam^1^, Gwangho Yoon^1,^ Eunhwa Ko^2^, Sang-Bum Kim^2^, Mahealani R. Bautista^3^, Christina C. Capule^3^, Takaoki Koyanagi^3^, Geoffray Leriche^3^, Hwan Geun Choi^2^, Jerry Yang^3^, Jeongyeon Kim^1,^*, Hyang-Sook Hoe^1,^*

^1^ Department of Neural Development and Disease, Korea Brain Research Institute (KBRI), 61 Cheomdan-ro, Dong-gu, Daegu, Korea, 41068; ^2^New Drug Development Center, Daegu-Gyeongbuk Medical Inovation Foundation, 80 Cheomdan-ro, Dong-gu, Daegu, Korea, 41061; ^3^ Department of Chemistry and Biochemistry, University of California San Diego, La Jolla, California, 92093-0358, USA

*Corresponding author

Hyang-Sook Hoe, Ph.D: Department of Neural Development and Disease, Korea Brain Research Institute (KBRI), 61 Cheomdan-ro, Dong-gu, Daegu, Korea, 41068

E-mail: [sookhoe72@kbri.re.kr](mailto:sookhoe72@kbri.re.kr)

Jeongyeon Kim, Ph.D: Department of Neural Development and Disease, Korea Brain Research Institute (KBRI), 61 Cheomdan-ro, Dong-gu, Daegu, Korea, 41068

E-mail: [jykim@kbri.re.kr](mailto:jykim@kbri.re.kr)

**Keywords**: Alzheimer’s disease, neuroinflammation, ERK, STAT3, LPS, CA140

**This PDF file includes:**

**Supplementary Figures 1-12 and Figure legends**

**
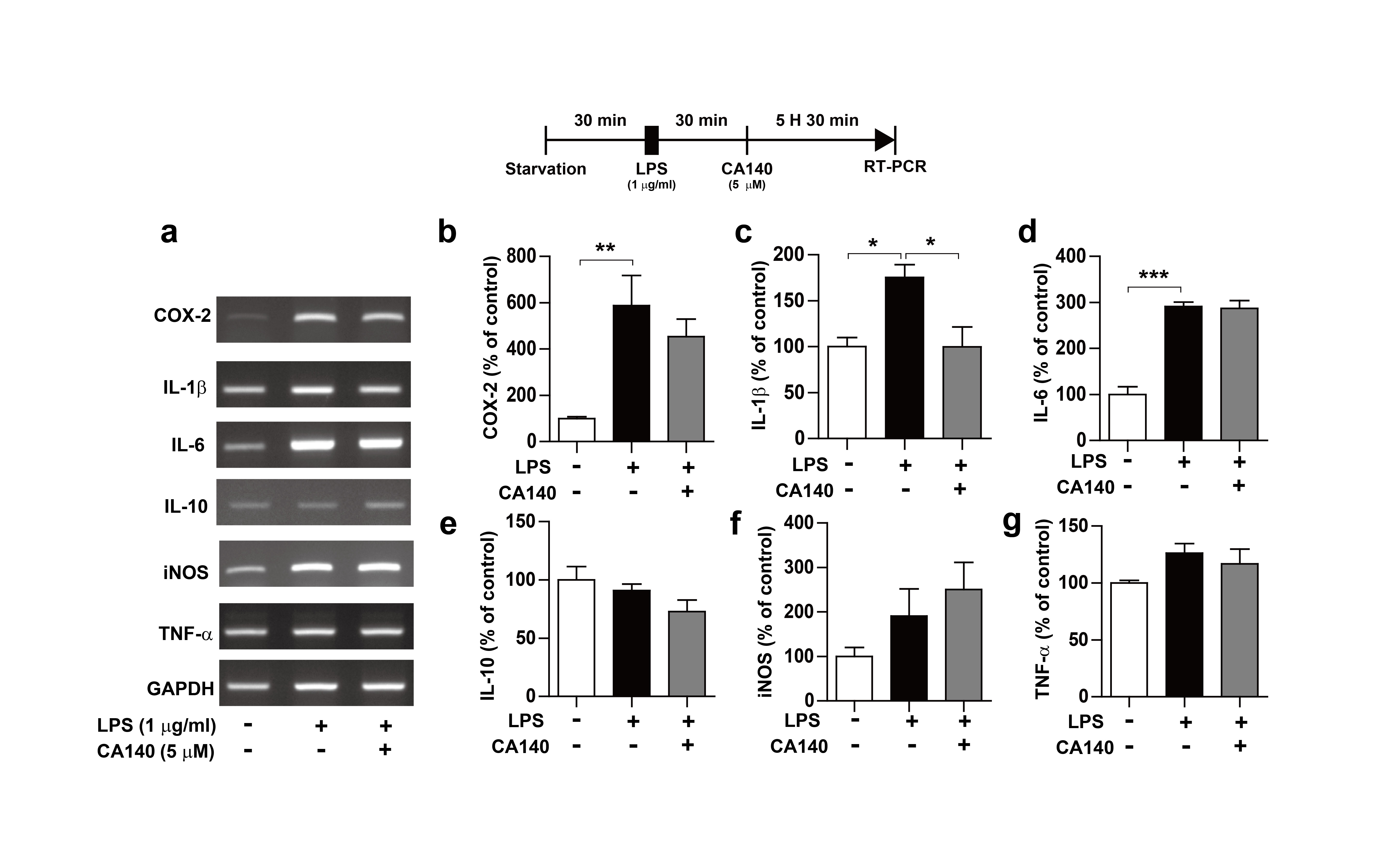
Figure S1** Post-treatment with CA140 at 5 μM only significantly reduced LPS-induced IL-1β mRNA levels. **a-g** BV2 microglial cells were pretreated with LPS (1 μg/ml) or PBS for 30 min and treated with vehicle (1% DMSO) or CA140 (5 μM) for 5 hr 30 min. Total RNA was isolated, pro-inflammatory cytokine levels were measured using RT-PCR (con, n=4; LPS, n=4; LPS+CA140, n=4). *p<0.05, **p<0.001, ***p<0.0001.

**
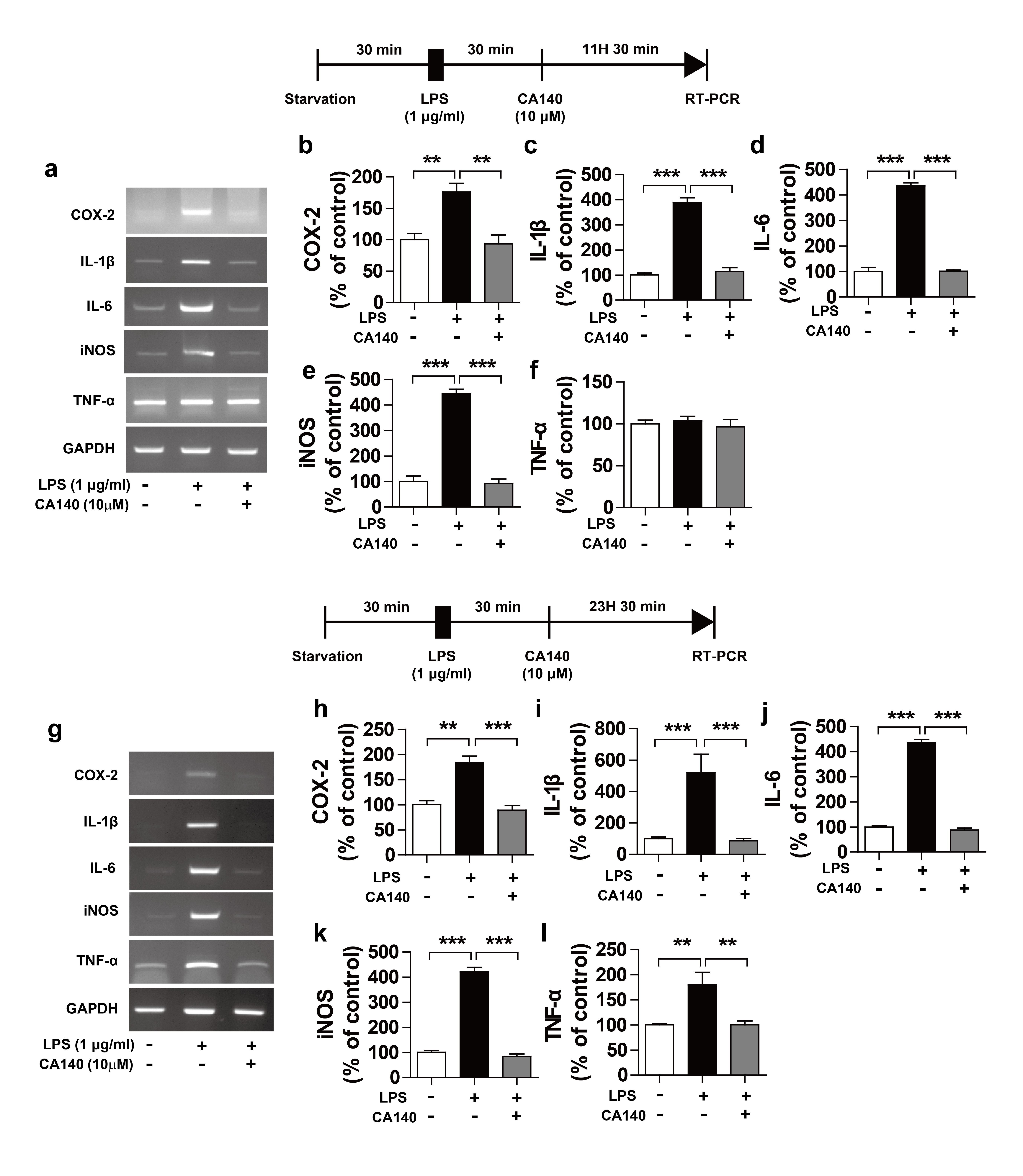
**

**Figure S2** Post-treatment with CA140 significantly reduced LPS-induced proinflammatory cytokine levels in a longer treatment. **a-f** BV2 microglial cells were pretreated with LPS (1 μg/ml) or PBS for 30 min and treated with vehicle (1% DMSO) or CA140 (10 μM) for 11 hr 30 min. Total RNA was isolated, pro-inflammatory cytokine levels were measured using RT-PCR (con, n=4; LPS, n=4; LPS+CA140, n=4). **g-l** BV2 microglial cells were pretreated with LPS (1 μg/ml) or PBS for 30 min and treated with vehicle (1% DMSO) or CA140 (10 μM) for 23 hr 30 min. Total RNA was isolated and pro-inflammatory cytokine levels were measured using RT-PCR (con, n=8; LPS, n=8; LPS+CA140, n=8). **p<0.001, ***p<0.0001.

**
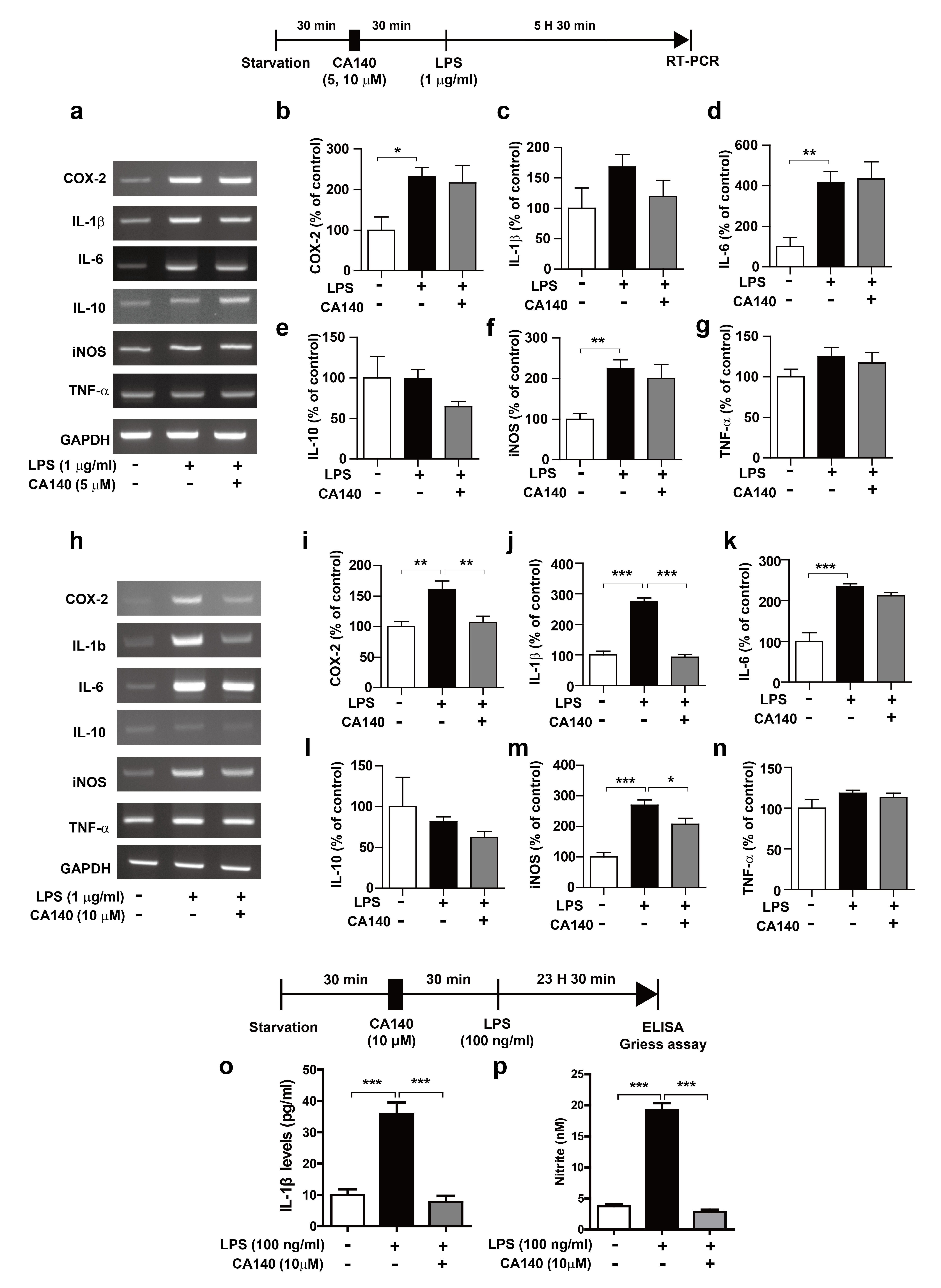
**

**Supplementary Fig. 3** Pre-treatment with CA140 significantly decreased LPS-induced COX-2, IL-1β, iNOS mRNA levels in BV2 microglial cells. **a-g** BV2 microglial cells were pretreated with vehicle (1% DMSO) or 5 μM CA140 for 30 min and treated with LPS (1 μg/ml) or PBS for 5 hr 30 min. Total RNA was isolated, and pro-inflammatory cytokines levels were measured using RT-PCR (con, n=4; LPS, n=4; LPS+CA140, n=4). **h-n** BV2 microglial cells were pretreated with vehicle (1% DMSO) or 10 μM CA140 for 30 min and treated with LPS (1 μg/ml) or PBS for 5 hr 30 min. Total RNA was isolated and pro-inflammatory cytokine levels were measured using RT-PCR (con, n=7; LPS, n=7; LPS+CA140, n=7). **o-p** BV2 microglial cells were pretreated with vehicle (1% DMSO) or CA140 (10 μM) for 30 min and treated with LPS (100 ng/ml) or PBS for 23 hr 30 min. The levels of IL-1β and NO were measured using IL-1β ELISA (con, n=8; LPS, n=8; LPS+CA140, n=8) or the Griess assay (to detect NO production; con, n=20; LPS, n=20; LPS+CA140, n=20). *p<0.05, **p<0.001, ***p<0.0001.


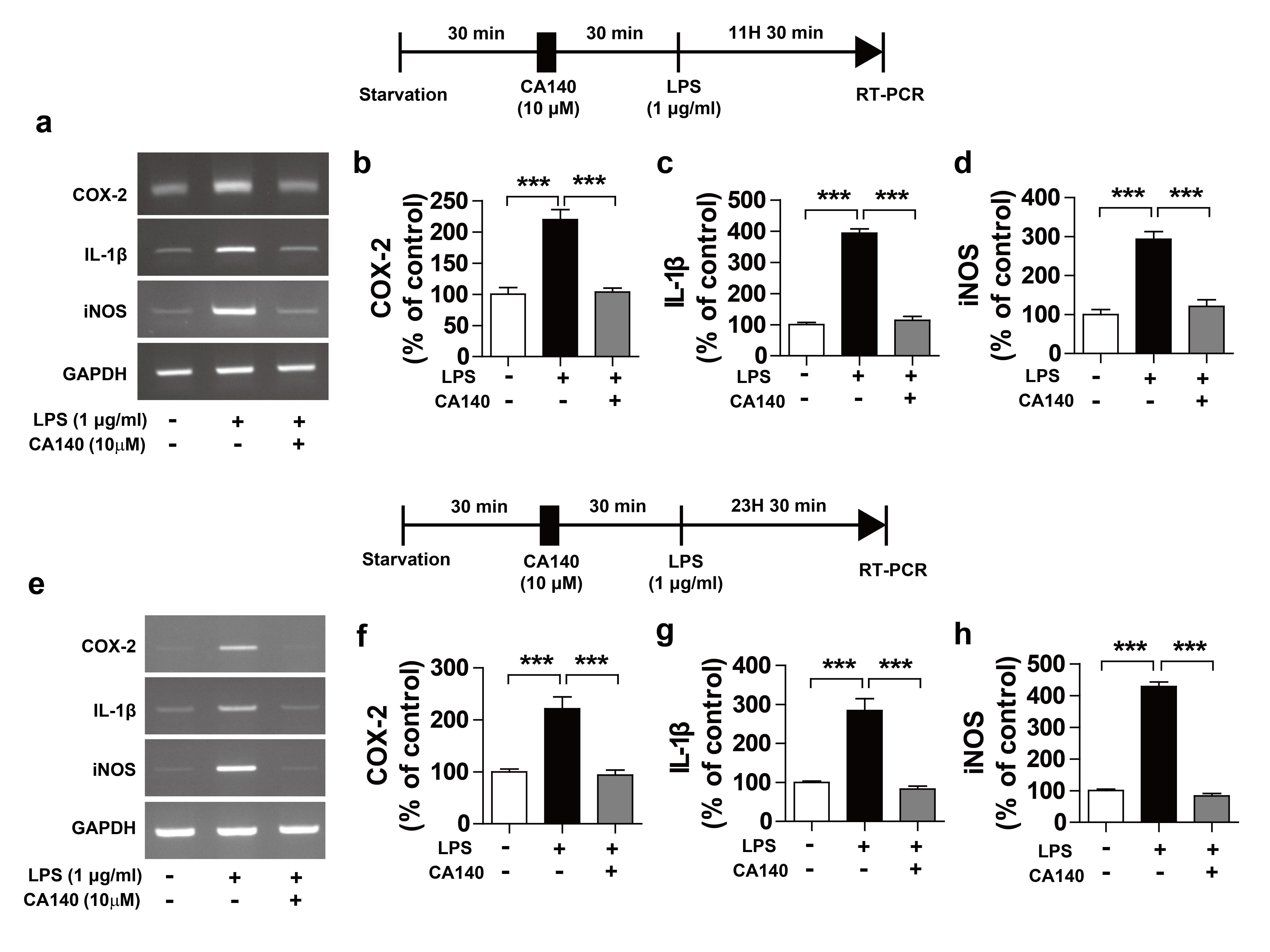


**Figure S4** Pre-treatment with CA140 significantly decreased LPS-induced COX-2, IL-1β, iNOS mRNA levels in a longer treatment. **a-d** BV2 microglial cells were pretreated with vehicle (1% DMSO) or CA140 (10 μM) for 30 min and treated with LPS (1 μg/ml) or PBS for 11 hr 30 min. Total RNA was isolated and pro-inflammatory cytokine levels were measured using RT-PCR (con, n=4; LPS, n=4; LPS+CA140, n=4). **e-h** BV2 microglial cells were pretreated with vehicle (1% DMSO) or CA140 (10 μM) for 30 min and treated with LPS (1 μg/ml) or PBS for 23 hr 30 min. Total RNA was isolated and pro-inflammatory cytokine levels were measured using RT-PCR (con, n=8; LPS, n=8; LPS+CA140, n=8). ***p<0.0001.


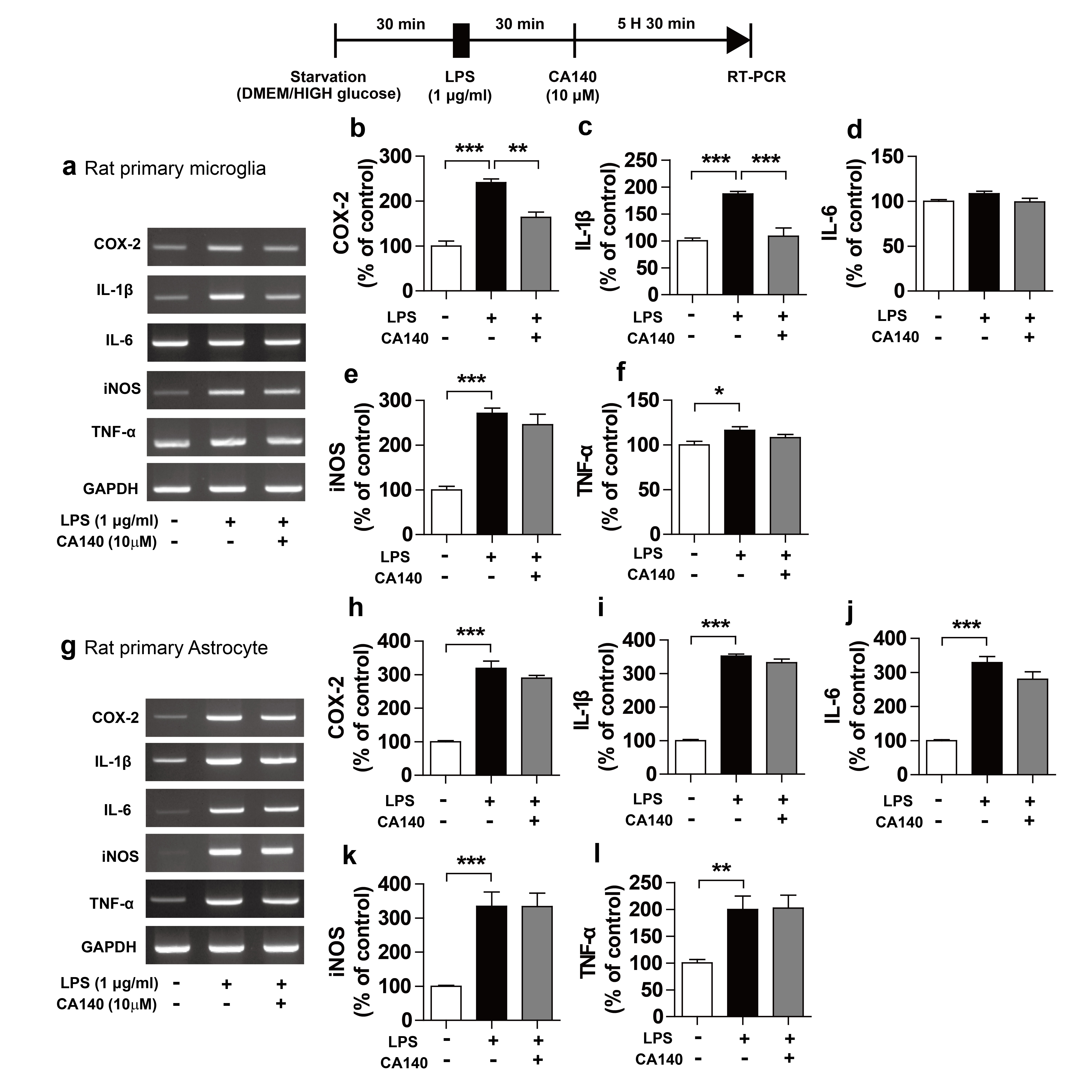


**Figure S5** Post-treatment with CA140 significantly decreased LPS-mediated proinflammatory cytokine levels in rat primary microglial cells. **a-f** Rat primary microglial cells were pretreated with LPS (1 μg/ml) or PBS for 30 min and treated with vehicle (1% DMSO) or CA140 (10 μM) for 5 hr 30 min. Total RNA was isolated and proinflammatory cytokine levels were measured using RT-PCR (COX-2, IL-1β, IL-6, iNOS, and TNF-alpha: con, n = 4; LPS, n = 4; LPS + CA140, n = 4). **g-l** Rat primary astrocytes were pretreated with LPS (1 μg/ml) or PBS for 30 min and treated with vehicle (1% DMSO) or CA140 (10 μM) for 5 hr 30 min. Total RNA was isolated and proinflammatory cytokine levels were measured using RT-PCR (COX-2, IL-1β, IL-6, iNOS, and TNF-alpha: con, n = 8; LPS, n = 8; LPS + CA140, n = 8). *p<0.05, **p<0.001, ***p<0.0001.

**
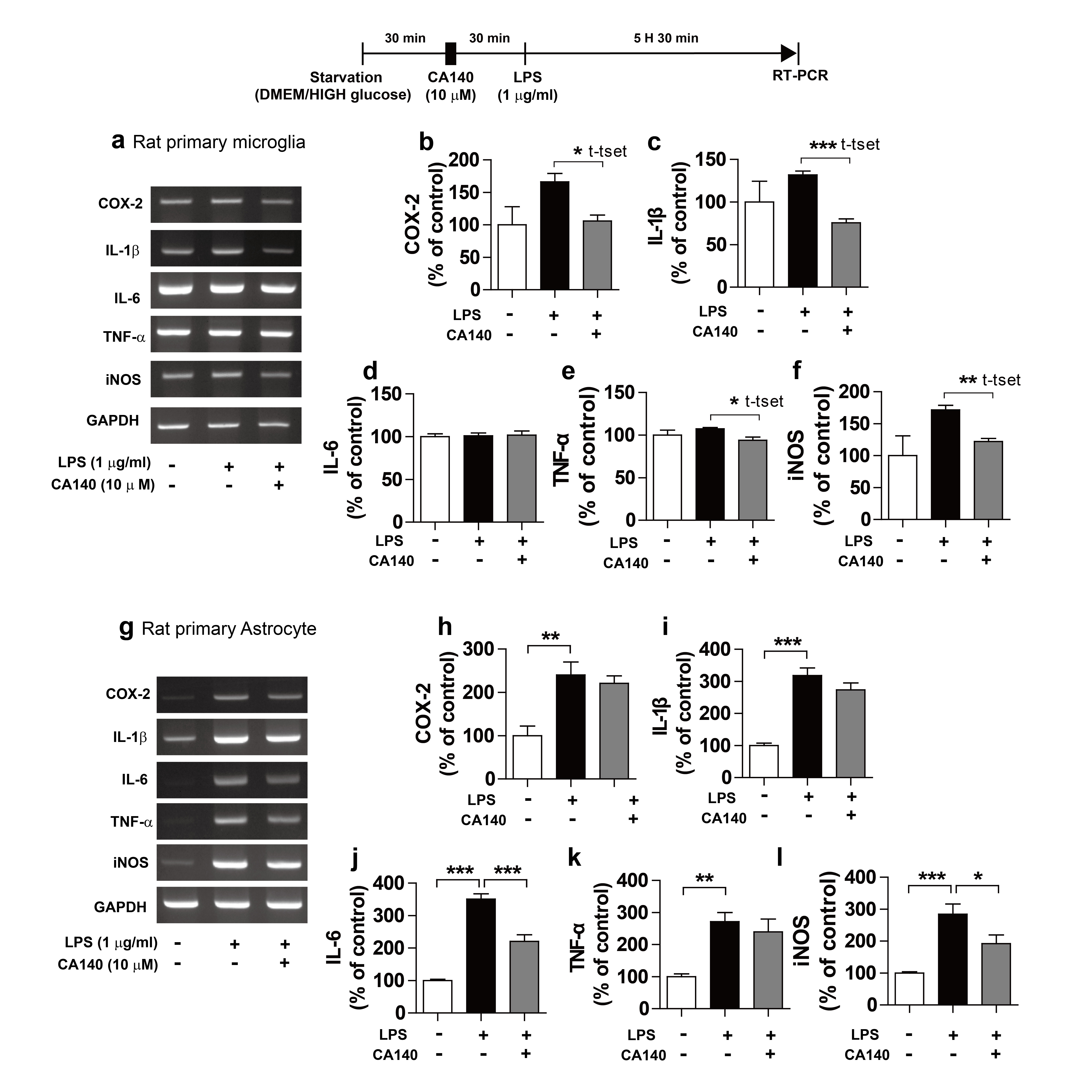
**

**Figure S6** Pre-treatment with CA140 decreased LPS-induced pro-inflammatory cytokine levels in rat primary microglial cells and primary astrocytes. **a-f** Rat primary microglial cells were pretreated with vehicle (1% DMSO) or CA140 (10 μM) for 30 min and treated with LPS (1 μg/ml) or PBS for 5 hr 30 min. Total RNA was isolated, and pro-inflammatory cytokine levels were measured using RT-PCR (con, n=4; LPS, n=4; LPS+CA140, n=4). **g-i** Rat primary astrocytes were pretreated with vehicle (1% DMSO) or CA140 (10 μM) for 30 min and treated with LPS (1 μg/ml) or PBS for 5 hr 30 min. Total RNA was isolated and pro-inflammatory cytokines levels were measured using RT-PCR (con, n=8; LPS, n=8; LPS+CA140, n=8). *p<0.05, **p<0.001, ***p<0.0001.


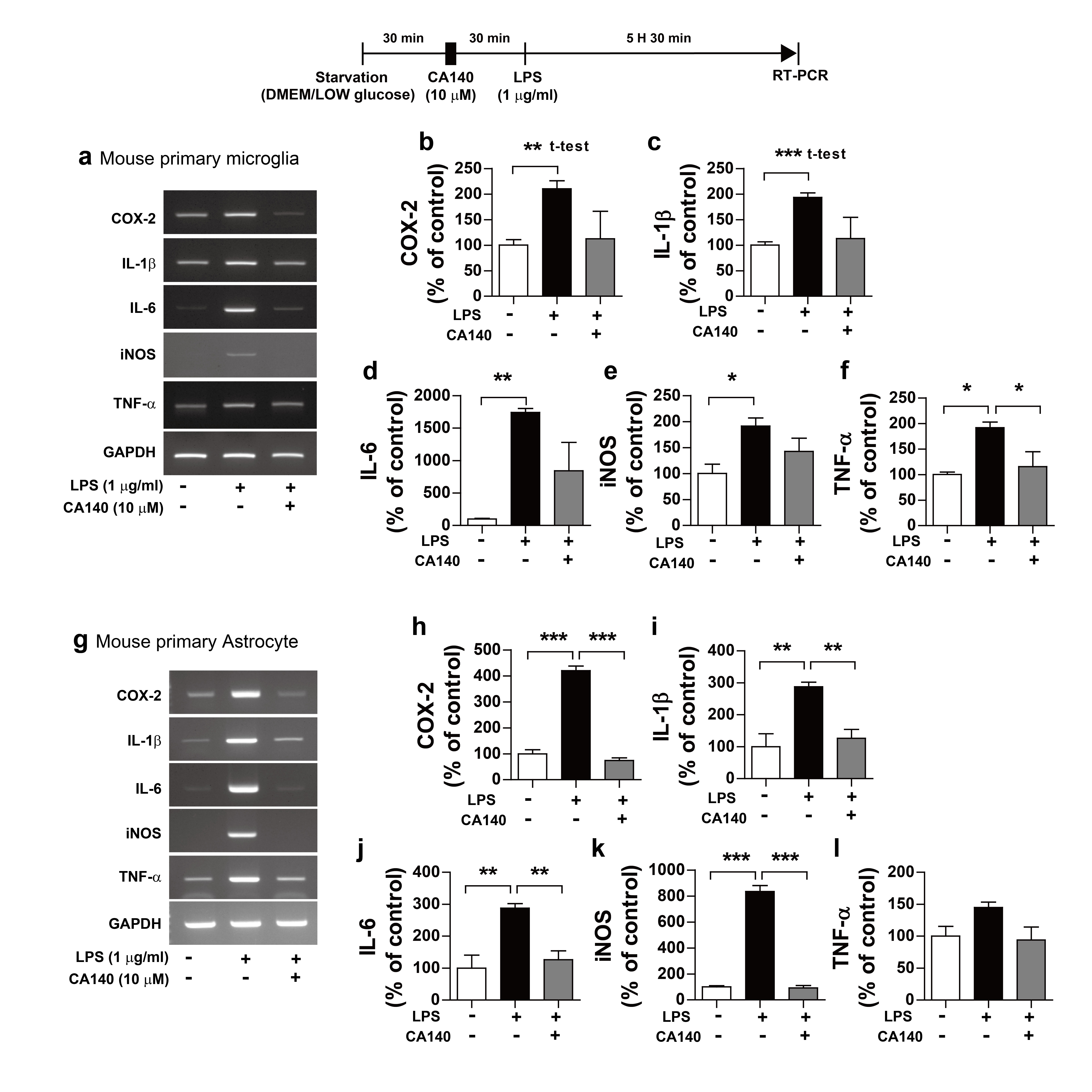


**Figure S7** Pre-treatment with CA140 decreased LPS-mediated pro-inflammatory cytokine levels in mouse primary microglial cells and primary astrocytes. **a-f** Mouse primary microglial cells were pretreated with vehicle (1% DMSO) or CA140 (10 μM) for 30 min and treated with LPS (1 μg/ml) or PBS for 5 hr 30 min. Total RNA was isolated and pro-inflammatory cytokine levels were measured using RT-PCR (con, n=4; LPS, n=4; LPS+CA140, n=4). **g-i** Mouse primary astrocytes were pretreated with vehicle (1% DMSO) or CA140 (10 μM) for 30 min and treated with LPS (1 μg/ml) or PBS for 5 hr 30 min. Total RNA was isolated and pro-inflammatory cytokines levels were measured using RT-PCR (con, n=4; LPS, n=4; LPS+CA140, n=4). *p<0.05, **p<0.001, ***p<0.0001.


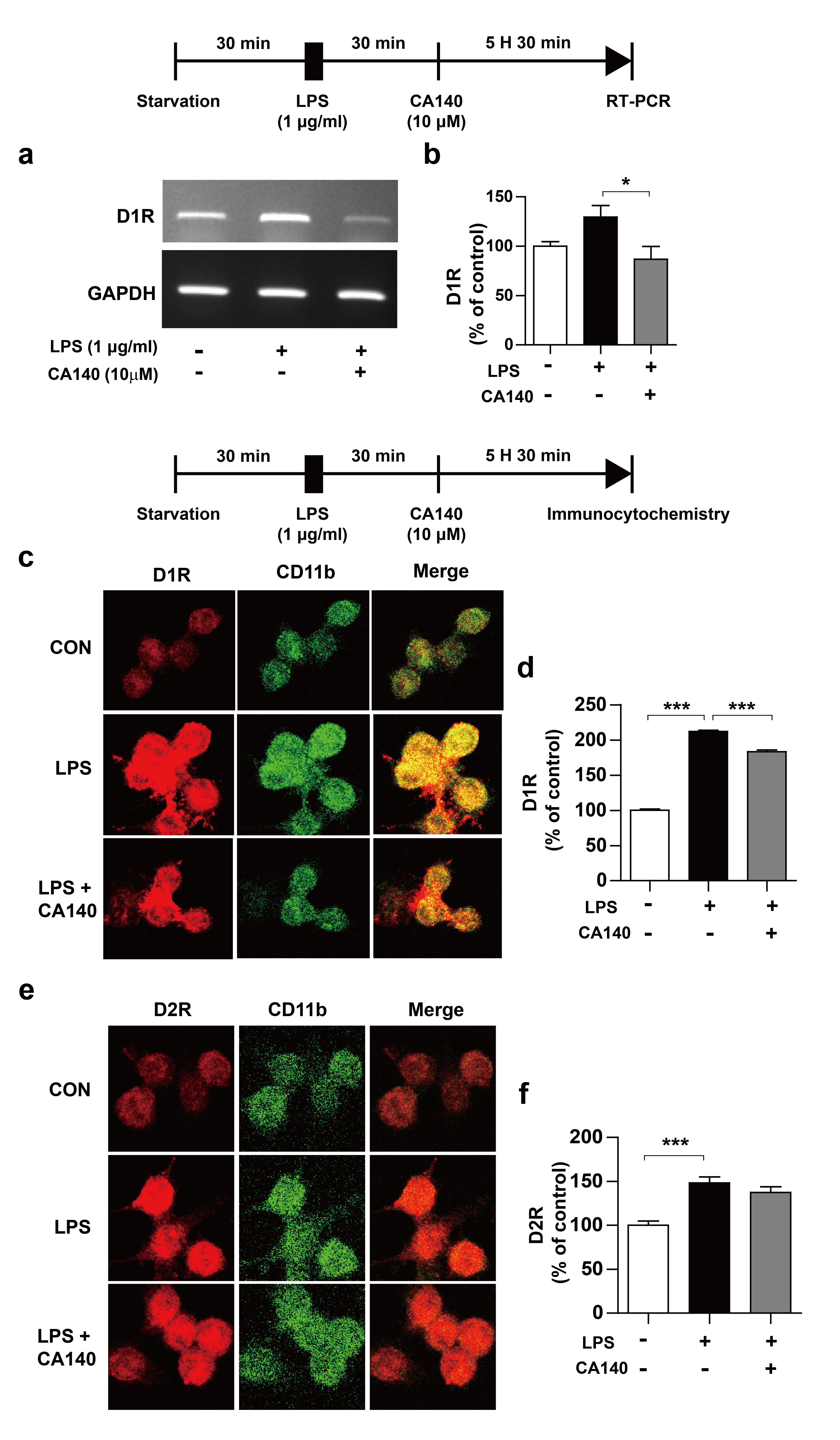


**Figure S8**. Post-treatment with CA140 downregulated LPS-induced dopamine D1 receptor (D1R) levels in BV2 microglial cells. **a-b** BV2 microglial cells were pretreated with LPS (1 μg/ml) or PBS for 30 min, treated with vehicle (1% DMSO) or CA140 (10 μM) for 5 hr 30 min. Total RNA was isolated and D1R levels were measured using RT-PCR (con, n=7; LPS, n=7; LPS+CA140, n=7). **c-d** BV2 microglial cells were pretreated with LPS (1 μg/ml) or PBS for 30 min, treated with vehicle (1% DMSO) or CA140 (10 μM) for 5 hr 30 min. Immunostaining were performed with anti-CD11b and anti-D1R antibodies (con, n = 643; LPS, n = 624; LPS + CA140, n = 489). **e-f** BV2 microglial cells were pretreated with LPS (1 μg/ml) or PBS for 30 min, treated with vehicle (1% DMSO) or CA140 (10 μM) for 5 hr 30 min. Immunostaining were performed with anti-CD11b and anti-D2R antibodies (con, n = 435; LPS, n = 489; LPS + CA140, n = 452). *p<0.05, ***p<0.0001.

**
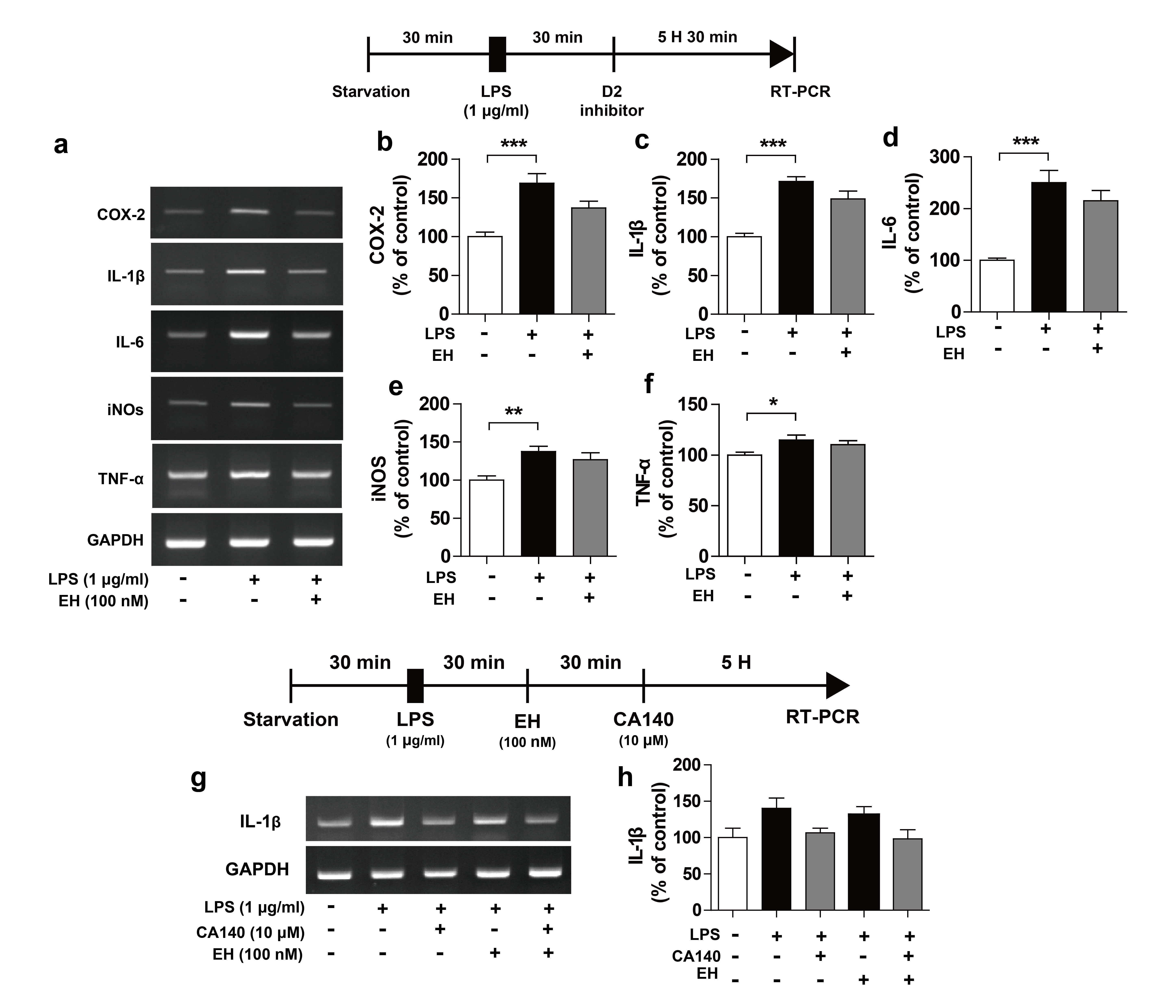
**

**Figure S9.** Inhibition of dopamine D2 receptor (D2R) did not reduce LPS-stimulated proinflammatory cytokine levels in BV2 microglial cells. **a-f** BV2 microglial cells were pretreated with LPS (1 μg/ml) or PBS for 30 min and treated with vehicle (1% DMSO) or Eticlopride hydrichloride (EH, D2R antagonist, 100 nM) for 5 hr 30 min. Total RNA was isolated and pro-inflammatory cytokine levels were measured using RT-PCR (COX-2, IL-1β, IL-6, iNOS, and TNF-alpha: con, n=12; LPS, n=12; LPS+CA140, n=12). **g-h** BV2 microglial cells were pretreated with LPS (1 μg/ml) or PBS for 30 min, treated with vehicle (1% DMSO) or EH (D2R antagonist, 100 nM) for 30 min, and treated with CA140 (10 μM) or vehicle (1% DMSO) for 5 hr. Total RNA was isolated and IL-1β mRNA levels were measured using RT-PCR (con, n=10; LPS, n=10; LPS+CA140, n=10). *p<0.05, **p<0.001, ***p<0.0001.

**
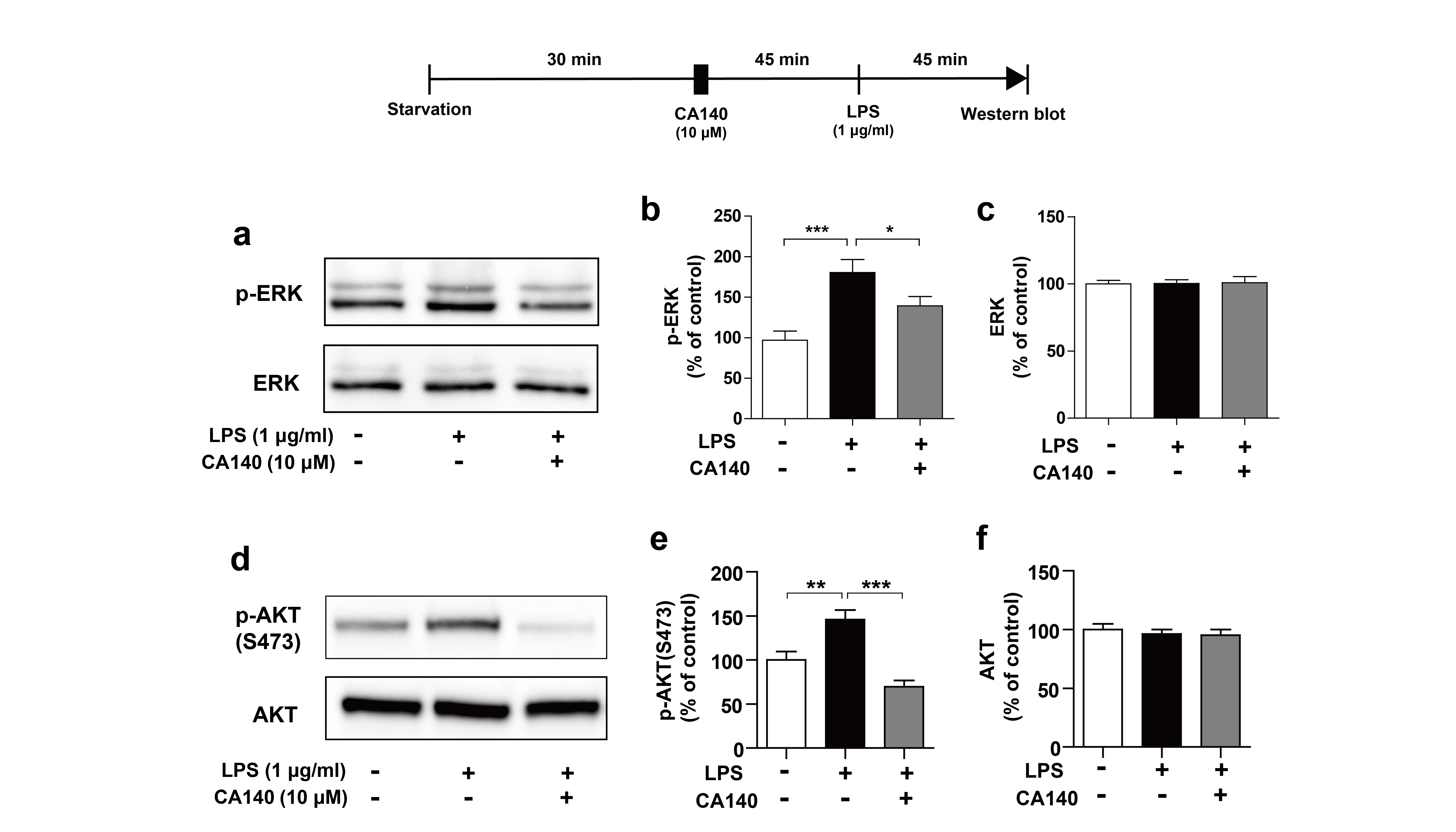
**

**Figure S10** Pre-treatment with CA140 significantly decreased phosphorylation of ERK and AKT in LPS-stimulated BV2 microglial cells. **a** BV2 microglial cells were pretreated with vehicle (1% DMSO) or CA140 (10 μM) for 45 min and treated with LPS (1 μg/ml) or PBS for 45 min. Cells were harvested and western blotting were conducted with anti-p-ERK and anti-ERK antibodies. **b-c** Quantification of data from **a** (p-ERK; con, n=19; LPS, n=19; LPS+CA140, n=19; ERK; con, n=23; LPS, n=23; LPS+CA140, n=23). **d** BV2 microglial cells were pretreated with vehicle (1% DMSO) or CA140 (10 μM) for 45 min and treated with LPS (1 μg/ml) or PBS for 45 min. Cells were harvested and western blotting were conducted with anti-p-AKT (Ser743) and anti-AKT antibodies. **e-f** Quantification of data from **d** (p-AKT and AKT; con, n=12; LPS, n=12; LPS+CA140, n=12). *p<0.05, **p<0.001, ***p<0.0001.

**
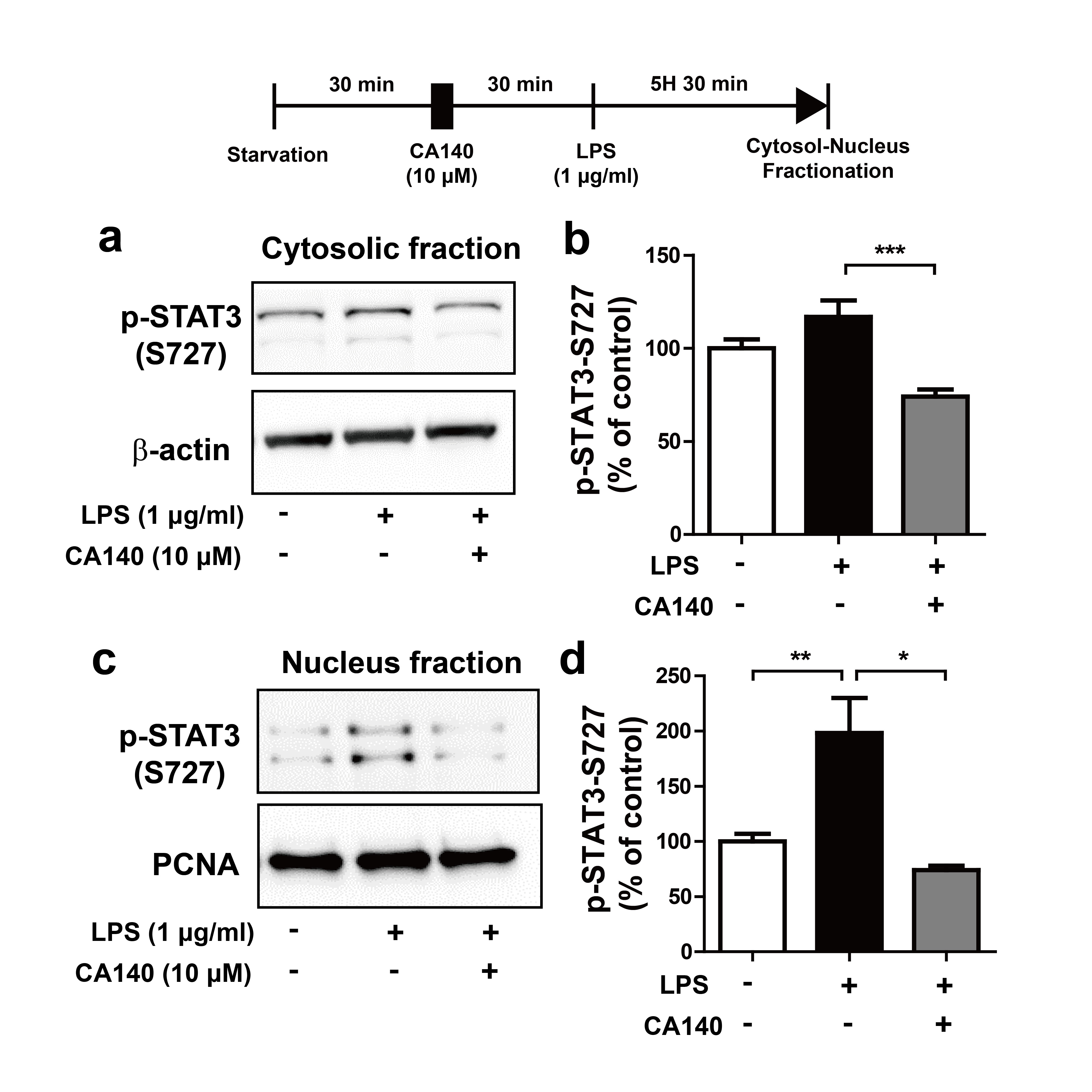
**

**Figure S11.** Pre-treatment with CA140 significantly decreased cytosolic and nuclear p-STAT3 levels in LPS-induced BV2 microglial cells. **a** BV2 microglial cells were pretreated with vehicle (1% DMSO) or CA140 (10 μM) for 30 min, treated with LPS (1 μg/ml) or PBS for 5hr 30 min, and then subjected to subcellular fractionation (nucleus vs. cytosol). Western blotting was performed on the cytosolic fraction using antibodies against anti-p-STAT3 (Ser727) and anti-β-actin. **b** Quantification of data from **a** (con, n=8; LPS, n=8; LPS+CA140, n=8). **c** Western blotting was performed on the nuclear fraction using antibodies against anti-p-STAT3 (Ser727) and anti-PCNA. **d** Quantification of data from **c** (con, n=8; LPS, n=8; LPS+CA140, n=8). *p<0.05, **p<0.001, ***p<0.0001.

**
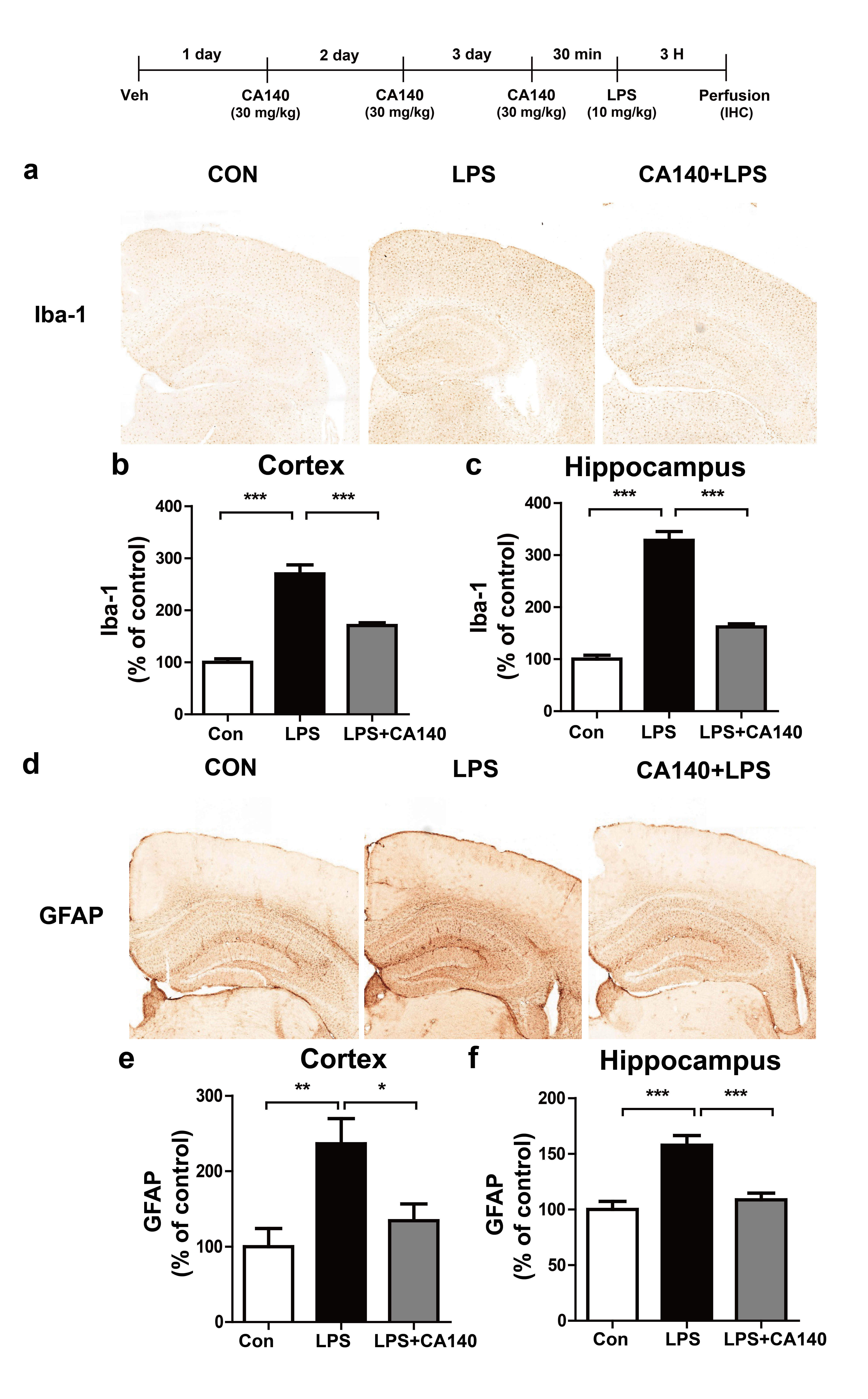
**

**Figure S12**. Pre-treatment with CA140 significantly reduced microglia and astrocyte activation in wild-type mice. **a** Wild-type mice were injected with CA140 (30mg/kg, i.p.) or vehicle (10% DMSO, i.p.) daily for 3 days, followed by injection with LPS (10mg/kg, i.p.) or PBS. Three hours later, immunohistochemistry were conducted with anti-Iba-1 antibody. **b-c** Quantification of data from **a** (con, n=5 mice; LPS, n=5 mice; LPS+CA140, n=5 mice). **d** Wild-type mice were injected with CA140 (30mg/kg, i.p.) or vehicle (10% DMSO, i.p.) daily for 3 days, followed by injection with LPS (10mg/kg, i.p.) or PBS. Three hours later, immunohistochemistry were conducted with anti-GFAP antibody. **e-f** Quantification of data from **d** (con, n=5 mice; LPS, n=5 mice; LPS+CA140, n=5 mice). *p<0.05, **p<0.001, ***p<0.0001.
